# Baicalin and valproic acid synergistically induce hepatocellular carcinoma cell apoptosis via ROS-mediated PTEN upregulation

**DOI:** 10.3389/fimmu.2026.1838349

**Published:** 2026-06-03

**Authors:** Zhiqiang Ni, Xianzhuo Jiang, Ying Zhou, Yingying Yu, Junjie Hou, Haifeng Wei, Jing Li

**Affiliations:** 1 Department of Medical Oncology, Jilin Provincial People's Hospital, Changchu, Jilin, China; 2 Neurobiology Laboratory, Wannan Medical College, Wuhu, Anhui, China

**Keywords:** apoptosis, baicalin, liver cancer cells, Pten, reactive oxygen species, valproic acid

## Abstract

**Background and objective:**

The prognosis of liver cancer remains exceedingly poor. The therapeutic potential of baicalin (BAI), a natural bioactive compound, in treating liver cancer is significantly limited by its low bioavailability. The histone deacetylase inhibitor valproic acid (VPA) has been shown to enhance the efficacy of anticancer agents. This study aims to investigate the synergistic antitumor effects and underlying molecular mechanisms of BAI and VPA in combination against liver cancer.

**Methods:**

*In vitro* experiments were conducted using the CCK-8 assay, morphological observation, AO/EB dual fluorescence staining, wound healing assay, transwell migration assay, and western blot analysis. These findings were further validated *in vivo* using a mouse subcutaneous syngeneic tumor model.

**Results:**

VPA alone exhibited no significant cytotoxicity against HepG2 and SMMC-7721 liver cancer cells, whereas BAI inhibited the proliferation of both cell lines in a concentration-dependent manner. The combination of BAI and VPA exerted stronger inhibitory effects on cell migration and greater promotion of apoptosis compared to either agent alone, with efficacy comparable to that of the chemotherapeutic drug doxorubicin. At the mechanistic level, the combined administration of drugs promotes the intracellular accumulation of reactive oxygen species (ROS), upregulates the expression of the PTEN protein, and activates the ROS-PTEN-Bax signaling axis to modulate the expression of apoptosis-related proteins, thereby ultimately inducing apoptosis in hepatocellular carcinoma cells. The synergistic pro-apoptotic effect was significantly reversed upon scavenging ROS with N-acetylcysteine or by knocking down PTEN expression, confirming a positive feedback regulatory relationship between ROS and PTEN. Further *in vivo* experiments consistently demonstrated that the combination treatment of BAI and VPA inhibits the growth of syngeneic tumors in a concentration-dependent manner, with the low-concentration combination achieving an antitumor efficacy comparable to that of high-concentration BAI monotherapy.

**Conclusion:**

BAI and VPA exert a synergistic anti-hepatocellular cancer effect through the ROS-PTEN-Bax pathway. This study provides a novel and effective therapeutic strategy for liver cancer and establishes a solid experimental foundation for repurposing VPA as a clinical adjuvant agent for BAI therapy.

## 1 Introduction

Liver cancer represents the most prevalent primary hepatic malignancy worldwide, with its pathogenesis and progression strongly associated with chronic viral hepatitis, liver cirrhosis, and other underlying liver disorders (1). As a leading cause of cancer-related mortality, liver cancer has maintained high global annual incidence and death rates for an extended period, posing a significant threat to public health. Owing to the absence of distinct early symptoms, the majority of patients are diagnosed at advanced stages, thereby missing the optimal window for curative interventions. Even among those who undergo potentially curative treatments such as surgical resection or liver transplantation, the high rate of postoperative recurrence remains a major clinical challenge. For patients with advanced or metastatic disease, tyrosine kinase inhibitors-including sorafenib and lenvatinib-serve as the cornerstone of conventional systemic therapy (2). However, these agents are limited by a low objective response rate, the frequent development of drug resistance, and considerable adverse effects, which collectively impair patients’ quality of life and hinder meaningful survival extension. Consequently, the 5-year overall survival rate remains unsatisfactorily low (3). Therefore, the development of novel therapeutic agents with improved efficacy and reduced toxicity, as well as the exploration of effective and synergistic combination treatment strategies, have become critical priorities in contemporary clinical research on liver cancer. Liver fibrosis is a critical pathological bridge between chronic liver injury and hepatocellular carcinoma (HCC), driving a progressive fibrosis-cirrhosis-cancer cascade and creating a pro-tumorigenic microenvironment that supports malignant transformation and metastasis. Recent large-scale cohort and mechanistic studies further confirm that fibrosis severity correlates with HCC risk and immune suppression, highlighting the necessity of targeting fibroinflammatory pathways in novel therapeutic designs (4). Notably, the pro-tumorigenic microenvironment driven by liver fibrosis is characterized by aberrant immune and inflammatory dysregulation, which is precisely a key trigger for HCC progression, immune escape, and therapeutic resistance.

The tumor immune and inflammatory microenvironment in HCC is characterized by persistent inflammation, immunosuppressive cell infiltration, and impaired anti-tumor immunity. These factors not only further exacerbate the progression of liver fibrosis but also collectively promote tumor progression, immune escape, and therapeutic resistance. Inflammatory cytokines and oxidative stress further amplify immune suppression and DNA damage, forming a self-reinforcing loop that accelerates malignant progression (5). Notably, baicalin (BAI) has been shown to regulate immune responses within the tumor microenvironment (6), while valproic acid (VPA) can enhance anti-tumor immunity by blocking myeloid-derived suppressor cell function (7), laying a theoretical foundation for the BAI-VPA combination to synergistically regulate the HCC immune microenvironment and break the vicious cycle between liver fibrosis and immune dysregulation.

The treasure trove of Traditional Chinese Medicine (TCM) harbors immense potential in the field of anti-tumor therapy. With the advancement of modern pharmacological research, the mechanisms underlying the therapeutic effects of bioactive components derived from TCM have been increasingly elucidated. BAI, a flavonoid glycoside extracted from medicinal herbs such as Scutellaria baicalensis Georgi (commonly known as Huangqin) and also present in Coptis chinensis, Coptis teeta, and Berberis species (commonly known as Three-needle), has a long history of clinical application in TCM and stands out as a highly valuable natural compound with diverse and significant biological activities. Notably, its anti-tumor properties are particularly prominent (8). These effects have been extensively validated across various models of solid and hematological malignancies, where BAI exerts its anti-cancer activity through synergistic modulation of multiple targets and signaling pathways. Specifically, it effectively suppresses tumor cell proliferation and clonogenic capacity, and induces cell cycle arrest. Furthermore, BAI triggers tumor cell apoptosis and autophagic cell death via both intrinsic and extrinsic pathways (9, 10). Concurrently, it downregulates key molecular mediators such as matrix metalloproteinases and vascular endothelial growth factor, thereby inhibiting tumor cell migration and angiogenesis (11, 12). BAI also demonstrates the ability to reverse multidrug resistance by modulating drug efflux transporters such as P-glycoprotein (P-gp) or by influencing apoptotic resistance pathways (13), and can regulate immune responses within the tumor microenvironment (6). However, BAI still encounters numerous bottlenecks that must be addressed to facilitate its clinical translation for anti-tumor therapy, significantly impeding the full realization of its therapeutic potential. First, extremely low oral bioavailability remains the primary challenge (14). Poor lipid solubility results in inefficient intestinal absorption, and the compound is prone to rapid degradation during hepatic first-pass metabolism. Additionally, P-gp-mediated drug efflux hinders the attainment of effective therapeutic concentrations in target organs-such as the liver-thereby directly compromising its anticancer efficacy. Second, the potential toxicity risks associated with BAI cannot be overlooked, which restricts the allowable clinical dosage. Finally, the therapeutic efficacy of monotherapy is inherently limited. Certain HCC cells exhibit insufficient sensitivity to BAI, and prolonged use of the agent alone may promote adaptive resistance in tumor cells, further diminishing treatment outcomes. Therefore, combination therapy strategies that achieve synergistic effects while mitigating toxicity have become a pivotal approach to overcoming the current clinical limitations of BAI and maximizing its antitumor potential. Compared with other anti-HCC natural products such as cinobufagin, which exerts potent anti-tumor effects but is limited by severe systemic toxicity and narrow therapeutic windows, BAI demonstrates favorable safety and multi-target activities; nevertheless, its monotherapy efficacy remains suboptimal (15). This comparison further highlights the necessity of developing combination strategies for BAI to enhance its efficacy while maintaining its safety advantage, which is the core objective of this study.

VPA, a well-established histone deacetylase inhibitor (16), has accumulated extensive safety data over decades of clinical use as an antiepileptic and mood-stabilizing agent, providing a robust foundation for its repurposing research as an anticancer therapeutic. The primary antitumor mechanism of VPA involves the specific inhibition of histone deacetylase activity, leading to a marked increase in the acetylation levels of both histone and non-histone proteins-such as transcription factors and molecular chaperones-within cells (17). Elevated histone acetylation promotes chromatin relaxation and reactivates the transcription of epigenetically silenced tumor suppressor genes. Meanwhile, acetylation of non-histone proteins modulates their stability, subcellular localization, and functional activity, thereby comprehensively reshaping the epigenetic landscape of tumor cells. This results in multiple anticancer effects, including induction of differentiation arrest, cell cycle arrest, apoptosis, and cellular senescence, while also significantly enhancing tumor cell sensitivity to radiotherapy, chemotherapy, and targeted therapies. Notably, VPA has demonstrated substantial synergistic effects with a broad range of antitumor agents-including chemotherapeutics, targeted drugs, and natural compounds-across various cancer models (7). For example, studies have shown that VPA enhances sorafenib-induced apoptosis in HCC cells through epigenetic reprogramming, via downregulation of apoptosis resistance pathways, suppression of DNA repair enzyme activity, and modulation of key signaling axes such as STAT3/NF-κB (18). Furthermore, VPA potentiates the cytotoxic effects of chemotherapeutic agents like doxorubicin (DOX) (19). These findings not only elucidate the multifaceted mechanisms underlying VPA’s synergistic antitumor activity but also offer strong theoretical support and promising translational potential for the combined application of BAI and VPA in liver cancer treatment.

Numerous natural compound-based nano-delivery systems have been developed for HCC therapy, primarily to improve the solubility, bioavailability, and tumor targeting of natural products, thereby addressing their inherent pharmacological limitations (20). However, such nano-formulation strategies face inherent challenges, including complex fabrication processes, difficulties in large-scale production, potential carrier-related toxicity, and high manufacturing costs. In contrast, the BAI-VPA combination strategy adopted in this study offers distinct advantages: it leverages two agents with established safety profiles (BAI with long-term TCM clinical application and VPA with decades of clinical use as an antiepileptic), avoids the risks of carrier toxicity and complex manufacturing, and simultaneously targets multiple key pathways (redox imbalance, epigenetic dysregulation, and immune microenvironment) in HCC. This makes our strategy simpler, more cost-effective, and more clinically translatable compared to nano-delivery systems, highlighting the innovative value of our research.

Reactive oxygen species (ROS) play a double-edged sword role in the regulation of cell fate (21). Notably, several clinical drugs cause liver injury via ROS overproduction and redox imbalance, while their toxicity and therapeutic windows are tightly linked to oxidative stress regulation-highlighting the importance of exploring agents that enhance anti-tumor ROS signaling while mitigating normal tissue damage (22). Tumor cells exhibit heightened sensitivity to ROS overload, and many chemotherapeutic and natural product-derived agents exert anti-tumor effects by inducing oxidative stress. BAI disrupts redox homeostasis in HCC cells by promoting ROS production and impairing antioxidant defenses, leading to lethal oxidative stress and cell death-consistent with our subsequent experimental findings that BAI induces ROS accumulation, which further mediates apoptotic signaling. Based on this mechanistic foundation, the present study systematically investigates whether BAI exerts its cytotoxic effects against liver cancer by inducing excessive ROS accumulation, and further elucidates the key signaling pathways involved in this process.

In response to the bottlenecks in the clinical translation of BAI for liver cancer therapy-such as low bioavailability and limited efficacy of monotherapy-this study systematically conducted *in vitro* cellular experiments and *in vivo* animal model validations. The objective was to elucidate the synergistic anti-HCC effects of the combination of BAI and VPA, thoroughly investigate the underlying molecular regulatory mechanisms mediated by ROS, and provide a robust experimental foundation and important theoretical support for the development of highly effective, low-toxicity combinatorial therapies for liver cancer. Furthermore, this work offers innovative insights into the clinical co-application of natural bioactive compounds and repurposed pharmaceuticals.

## 2 Materials and methods

### 2.1 Cell culture

The human HCC cell lines HepG2 and SMMC-7721 were purchased from Cell Bank of Type Culture Collection of Chinese Academy of Sciences (CAS), Shanghai, China. Cells were maintained in Dulbecco’s Modified Eagle Medium (DMEM, Gibco, Thermo Fisher Scientific, USA) supplemented with 10% fetal bovine serum (FBS, Gibco, Thermo Fisher Scientific, USA) and 1% penicillin-streptomycin (HyClone, USA). Cells were incubated in a humidified atmosphere containing 5% CO_2_ at 37°C to ensure optimal growth conditions. When the cultures reached 80-90% confluence, cells were passaged using trypsin-EDTA solution (Gibco, Thermo Fisher Scientific, USA) for detachment and subculturing. Routine monitoring of cell morphology and growth status was performed under a phase-contrast microscope. The culture medium was refreshed every 2–3 days to maintain consistent nutrient availability and optimal culture conditions.

### 2.2 Cell viability assay

In this study, cell viability was evaluated using the cell counting kit-8 (CCK-8, Dojindo Molecular Technologies, Japan) assay. Cells were seeded into 96-well plates (Corning, USA) at a density of 5,000 cells per well. Subsequently, cells were treated with various concentrations of BAI, VPA, or their combination. After incubation for 24 h and 48 h, the culture medium was removed, and 10% (v/v) fresh CCK-8 solution was added to each well. The plates were incubated at 37°C for 30 min, followed by measurement of absorbance at 450 nm using a microplate reader. Cell viability was determined by normalizing the absorbance values of treated groups to those of the control group. All experiments were performed in triplicate.

### 2.3 Cell morphological analysis

For morphological analysis, HepG2 and SMMC-7721 cells were seeded in 6-well plates (Corning, USA) at a density of 1×10^5^ cells per well and cultured in DMEM supplemented with 10% FBS until reaching 80% confluence. Subsequently, cells were treated with complete medium alone (negative control [NC]), BAI-containing medium (BAI group), VPA-containing medium (VPA group), or medium containing both agents (Com group). After 24 h of incubation, the medium was discarded, and the cells were gently rinsed twice with phosphate-buffered saline (PBS, Solarbio, Beijing, China). Cellular morphology was observed and imaged under an inverted phase-contrast microscope. Changes in cell adhesion capacity, and intracellular granules were documented and compared between the treatment groups and the control group.

### 2.4 Cell scratch healing and migration experiments

In this study, wound healing assays and transwell (Corning, USA) migration assays were employed to assess the migratory capacity of HepG2 and SMMC-7721 cells. Cells were seeded into six-well plates (Corning, USA) and cultured to confluence to form a uniform monolayer. A sterile pipette tip was used to create consistent linear scratches across the cell monolayer. Subsequently, the cells were gently washed with PBS to remove non-adherent cells and cellular debris, followed by treatment with BAI, VPA, or a combination of BAI and VPA. The cells were then incubated in a humidified atmosphere at 37°C with 5% CO_2_, and the migration and wound closure dynamics were monitored at 24 h and 48 h post-scratching. Microscopic images of the scratched areas were acquired at each time point, and the scratch width was measured over time to calculate the wound healing rate, thereby evaluating the cellular migration rate.

The migration assay was performed using transwell chambers. A 200 μL cell suspension at a concentration of 1×10^6^ cells/mL was seeded into the upper chamber, which contained serum-free medium; the lower chamber was filled with complete culture medium supplemented with BAI, VPA, or their combination treatments. After an incubation period of 24 h, non-migrated cells remaining on the upper surface of the membrane were gently removed with a cotton swab. The migration experiment was continued for a total duration of 48 h. Subsequently, cells that had migrated through the membrane pores and adhered to the lower surface were fixed with 4% paraformaldehyde (Solarbio, Beijing, China) and stained with crystal violet (Sigma-Aldrich, USA). Finally, the stained cells were visualized and counted under a microscope. Cell migratory capacity was quantified by counting cells across multiple fields of view. All experiments were independently repeated three times.

### 2.5 Detection of apoptosis

In this study, the acridine orange (AO)/ethidium bromide (EB) dual fluorescence staining kit (Solarbio, Beijing, China) was employed to evaluate cellular apoptosis. Following treatment with BAI, VPA, and their combinations, cells were harvested and washed with PBS. Subsequently, cells were stained with a freshly prepared working solution containing both AO and EB. The stained cells were immediately examined under a fluorescence microscope using appropriate excitation filters. Viable cells exhibited bright green fluorescence due to AO intercalation into double-stranded DNA, whereas apoptotic cells displayed orange-red fluorescence as a result of EB uptake through compromised membrane integrity. The percentage of apoptotic cells relative to the total cell population was determined by analyzing multiple random fields of view. All experiments were performed in triplicate.

### 2.6 Comparison of DOX’s antitumor effects

To assess the antitumor efficacy of the combined BAI and VPA treatment, DOX (Sigma-Aldrich, USA) was used as the positive control in this study. The cytotoxic effects of the BAI and VPA combination, in comparison to DOX, were systematically evaluated in liver cancer models using the CCK-8 assay, morphological assessment, and AO/EB staining for apoptosis detection.

### 2.7 ROS detection

Intracellular ROS levels were assessed using dihydroethidium (DHE, Sigma-Aldrich, USA) staining. Cells were seeded into six-well plates and treated with BAI, VPA, or their combination for 6 h. Following treatment, cells were gently washed with pre-warmed PBS and then incubated with 5 μM DHE solution in the dark at 37°C for 30 min. Excess dye was subsequently removed by washing with PBS. Fluorescence images were acquired using a fluorescence microscope equipped with appropriate filters, with a fixed exposure time of 100 ms. Fluorescence intensity was quantitatively analyzed using ImageJ software, and the mean fluorescence intensity for each treatment group was calculated. All experiments were performed in triplicate to ensure reproducibility.

### 2.8 Western blot analysis

Following drug treatment, HepG2 cells were lysed with lysis buffer (Beyotime, Shanghai, China) supplemented with 1% phenylmethylsulfonyl fluoride (Beyotime, Shanghai, China). Protein concentrations were subsequently determined using a bicinchoninic acid protein assay kit (Thermo Fisher Scientific, USA). Equal amounts of protein samples were separated by sodium dodecyl sulfate-polyacrylamide gel electrophoresis and then transferred onto polyvinylidene fluoride membranes (Millipore, USA). The membranes were blocked to prevent non-specific binding and incubated overnight at 4°C with primary antibodies (Cell Signaling Technology, CST, USA) specific to the target proteins, including phosphatase and tensin homolog deleted on chromosome ten (PTEN), B-cell lymphoma-2 (Bcl-2), Bcl-2-associated X protein (Bax), cleaved cysteinyl aspartate specific proteinase-3 (cleaved-caspase-3), cleaved poly (ADP-ribose) polymerase (cleaved-PARP), epithelial cadherin (E-cadherin), vimentin, snail, slug, and neuronal cadherin (N-cadherin). After thorough washing, horseradish peroxidase-conjugated secondary antibodies (ZSGB-BIO, Beijing, China) were applied and incubated at room temperature for 1–2 h. Protein bands were visualized using an enhanced chemiluminescence detection system (Millipore, USA), and band intensities were quantified using ImageJ software. To ensure accurate normalization and data reliability, GAPDH (Cell Signaling Technology, CST, USA) was used as an internal loading control.

### 2.9 Rescue experiment

To further investigate the role of ROS in enhancing the therapeutic efficacy of the combined treatment, N-acetylcysteine (NAC, Sigma-Aldrich, USA) was employed as a ROS inhibitor. Specifically, HepG2 cells were cultured in NC group, Com group, or medium containing BAI, VPA, and NAC (the Res group). Subsequently, ROS levels were measured to confirm the effectiveness of ROS inhibition. The rescue effect was then evaluated using the CCK-8 assay and cell apoptosis analysis, while the expression of relevant genes and proteins was assessed by RT-qPCR and western blotting. This comprehensive approach enabled a systematic evaluation of the impact of ROS inhibition on the outcomes of the combined treatment strategy.

To validate the involvement of the PTEN-Bax pathway in the apoptosis induced by the combined action of BAI and VPA, a PTEN rescue experiment was performed. The procedure was as follows: HepG2 cells were cultured in serum-free medium until they reached 70-80% confluence. A complex of si-PTEN (GenePharma, Shanghai, China) and Lipofectamine 2000 reagent (Invitrogen, Thermo Fisher Scientific, USA) was prepared according to the manufacturer’s instructions: briefly, 50 pmol si-PTEN and 2 μL Lipofectamine 2000 were separately diluted in 50 μL serum-free DMEM, incubated at room temperature for 5 min, then mixed and incubated for another 10 min to form a stable transfection complex. After incubation at 25 °C for 15 min, the mixture was added to the HepG2 cell culture medium and incubated for 24 h to ensure efficient transfection. This protocol successfully facilitated the delivery of si-PTEN into HepG2 cells. A non-targeting siRNA (si-NC) was used as a negative control to exclude non-specific effects of transfection. After transfection, the knockdown efficiency of PTEN was verified by RT-qPCR and Western blot analysis to ensure successful PTEN silencing. Subsequently, three experimental groups were established: the NC group, the Com group, and the si-PTEN group. Finally, the functional rescue following PTEN knockdown was assessed through CCK-8 assays, AO/EB staining, ROS detection, and western blot analysis.

### 2.10 *In vivo* anti-tumor experiments

In the *in vivo* anti-tumor studies, Kunming mice and H22 hepatoma cell line were purchased from Laboratory Animal Center of Jilin University, Changchun, China. All animal procedures were conducted in strict accordance with the Guide for the Care and Use of Laboratory Animals published by the National Institutes of Health of the United States and were approved by Laboratory Animal Ethics Committee of Wannan Medical College. The experimental mice were randomly assigned to four groups: the NC group, the BAI group, the VPA group, and the Com group. Subcutaneous tumors were established by injecting murine hepatoma H22 cells into the right flank of each mouse. BAI (purity ≥98%, Yuanye Biotechnology, Shanghai, China) and VPA (Sigma-Aldrich, USA) were used in this study. The treatment regimen involved administering 50 mg/kg of BAI, 300 mg/kg of VPA, or their combination to the respective groups. All treatments were delivered via intraperitoneal injection once every three days over a two-week period.

Throughout the experiment, tumor growth was monitored regularly using calipers, and tumor volumes were calculated based on the measurements. At the end of the treatment period, mice were humanely euthanized, and tumor tissues were harvested for further analysis. The anti-tumor efficacy of BAI, VPA, and their combination was comprehensively evaluated by comparing key parameters such as tumor growth inhibition rates and final tumor volumes across the different treatment groups. This experimental approach enabled a robust assessment of the *in vivo* inhibitory effects of these treatments on liver cancer progression.

## 3 Results

### 3.1 Synergistic cytotoxicity of BAI and VPA in liver cancer cells

**Figure 1:**
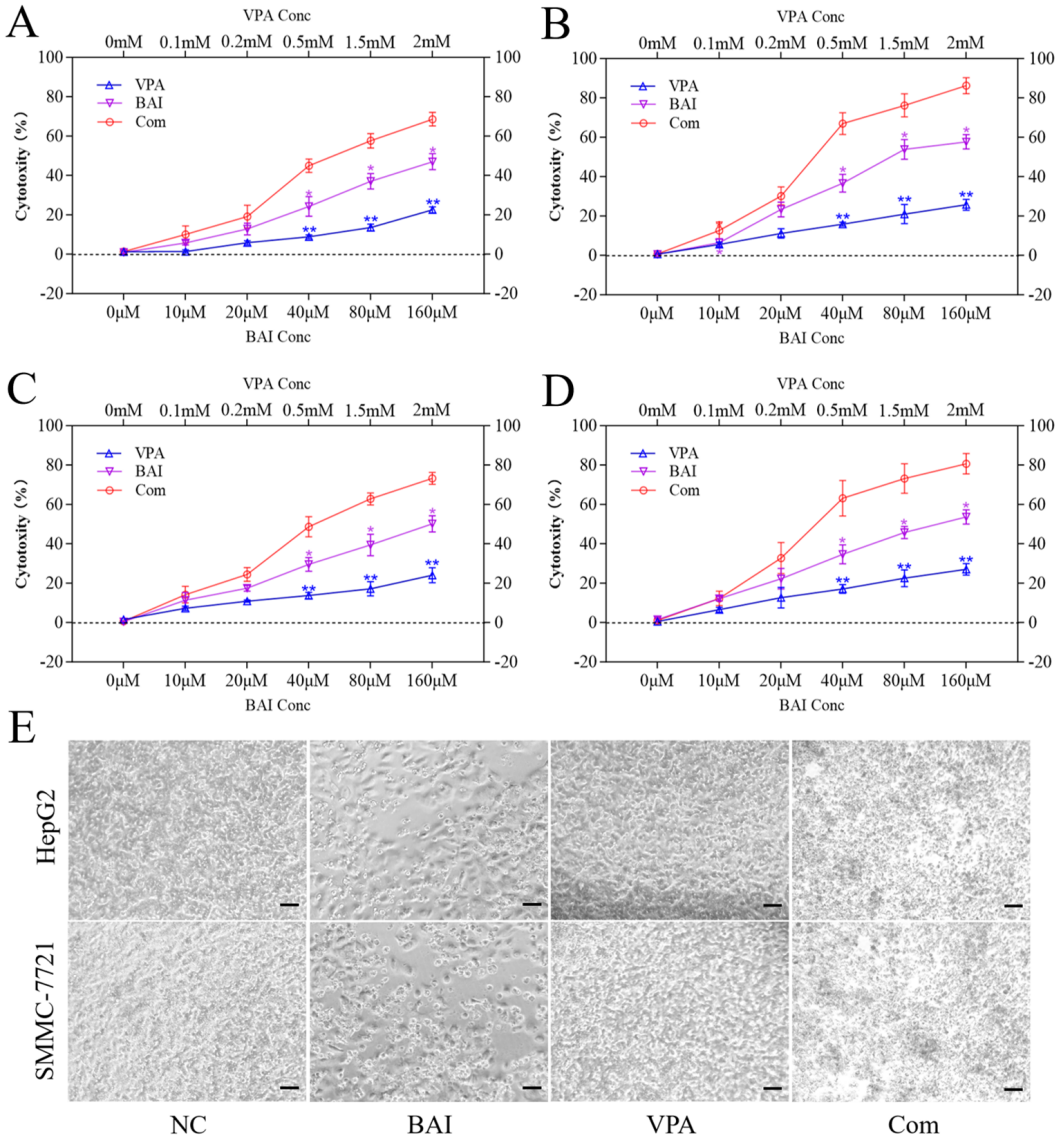
BAI and VPA exert a synergistic effect in eliminating liver cancer cells. HepG2 **(A, B)** and SMMC-7721 **(C, D)** cells were treated with increasing concentrations of BAI, VPA, or their combination (Com) for 24 h **(A, C)** or 48 h **(B, D)**. Cell viability was subsequently evaluated using the CCK-8 assay. **(E)** Microscopic observation of the morphological changes in liver cancer cell lines HepG2 and SMMC-7721 following treatment with BAI (40 μM) and/or VPA (0.5 mM) for 24 h. NC, Negative control; BAI, Baicalin; VPA, Valproic acid; Com, BAI+VPA. Scale bar, 100 μm. *compared with the Com group; *p < 0.05; **p < 0.01.

CCK-8 assay results demonstrated that VPA alone, at all tested concentrations, failed to elicit significant cytotoxicity in HepG2 (**Figures 1A, B**) or SMMC-7721 (**Figures 1C, D**) cells following 24 h (**Figures 1A, C**) or 48 h (**Figures 1B, D**) of incubation, with cell viability consistently maintained above 80%. In contrast, BAI exhibited a prominent concentration-dependent cytotoxic effect against both liver cancer cell lines. For example, after treatment with 40 μM BAI for 24 h, cell viability decreased to approximately 70% in both HepG2 and SMMC-7721 cells, while at the highest concentration (160 μM), viability dropped below 50% in both cell lines. Notably, co-administration of VPA with BAI significantly enhanced the cytotoxicity of BAI at the same concentration. When combined with 0.5 mM VPA, 40 μM BAI treatment for 24 h further reduced cell viability to approximately 40% in both cell lines, representing an approximately 2-fold increase in cytotoxicity compared with BAI monotherapy at the same concentration. These findings suggest that VPA may potentiate the cytotoxic activity of BAI in liver cancer cells. Further analysis revealed that the combination of 0.5 mM VPA and 40 μM BAI exerted a marked synergistic cytotoxic effect on HepG2 and SMMC-7721 cells at the 24 h time point, with a clear synergistic enhancement and no significant toxicity observed for VPA alone. Consequently, the 24 h incubation period, along with BAI at 40 μM and VPA at 0.5 mM, was selected as the optimal condition for subsequent experiments.

As shown in **Figure 1E**, observations under an inverted bright-field microscope revealed significant differences in cell density and optical properties of HepG2 and SMMC-7721 HCC cells among different treatment groups. Cells in the NC group and VPA group maintained normal morphological characteristics with strong adhesion and abundant cell numbers, and no abnormal deposits were observed in the field of view. In the BAI group, partial cells exhibited morphological abnormalities and weakened adhesion capacity, presenting a suspended state, accompanied by granular deposits induced by cell damage and metabolic disorders (23). The Com group showed a marked reduction in cell adhesion ability, along with extensive cell suspension and shedding, as well as a sharp increase in granular deposits. Overall, these results visually demonstrated that the combined treatment of BAI and VPA exerted a synergistic cytotoxic effect on HCC cells, which was consistent with the aforementioned CCK-8 assay results.

### 3.2 VPA enhances the anti-migratory effect of BAI on liver cancer cells

**Figure 2:**
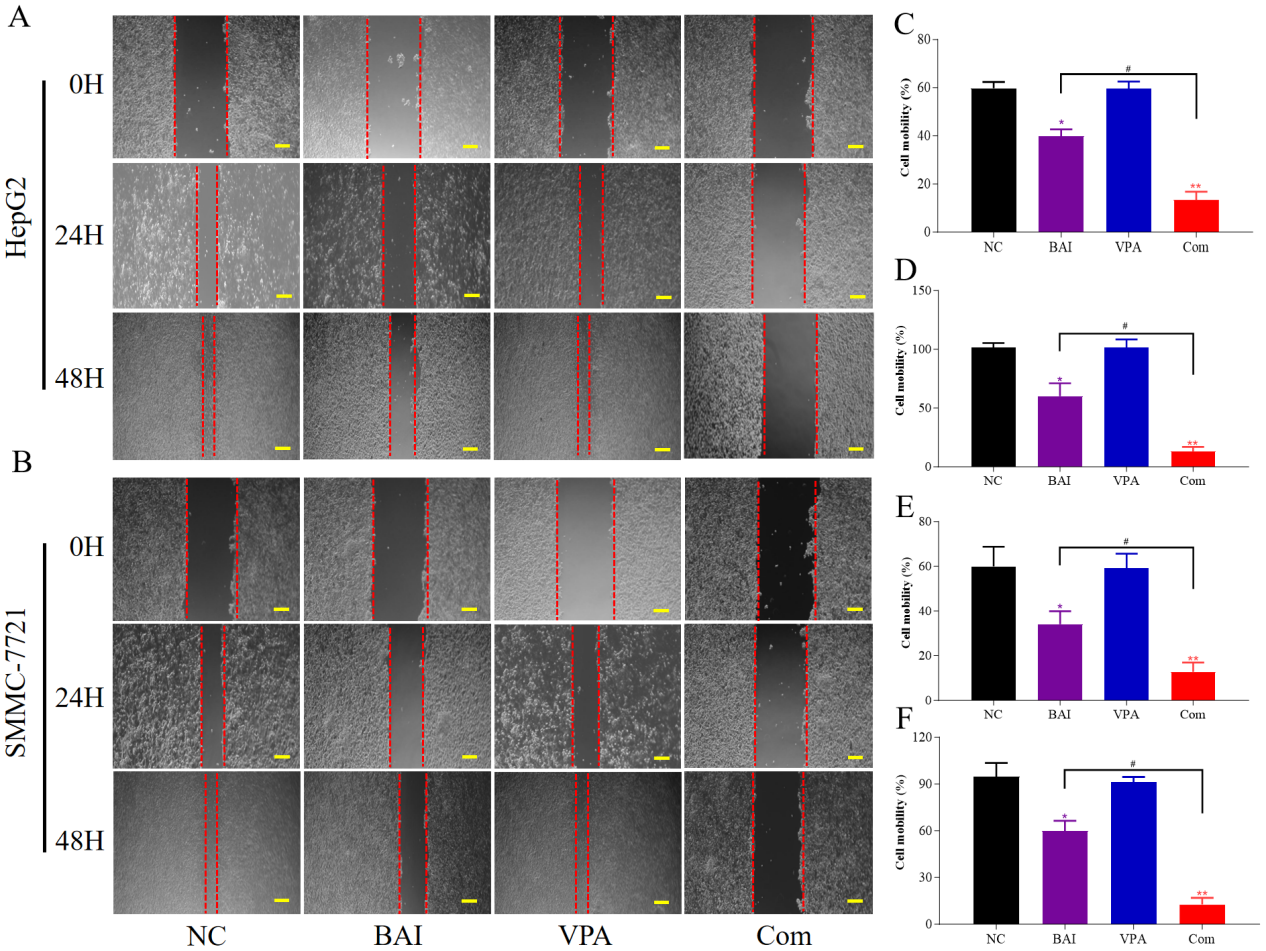
BAI and VPA inhibit the migratory ability of liver cancer cells. **(A, B)** Representative images of the scratch wound healing assay. HepG2 **(A)** and SMMC-7721 **(B)** cells were treated with BAI (40 μM), VPA (0.5 mM), or their combination (Com), and the wound closure was photographed at 0 h, 24 h, and 48 h. Scale bar, 100 μm. **(C-F)** Quantitative analysis of the wound healing rate. The relative wound closure percentage was calculated for HepG2 cells at 24 h **(C)** and 48 h **(D)**, and for SMMC-7721 cells at 24 h **(E)** and 48 h **(F)**. NC, Negative control; BAI, Baicalin; VPA, Valproic acid; Com, BAI+VPA. *, compared with the NC group; *p < 0.05; **p < 0.01; ^#^compared with the Com group; ^#^p < 0.05.

**Figure 3:**
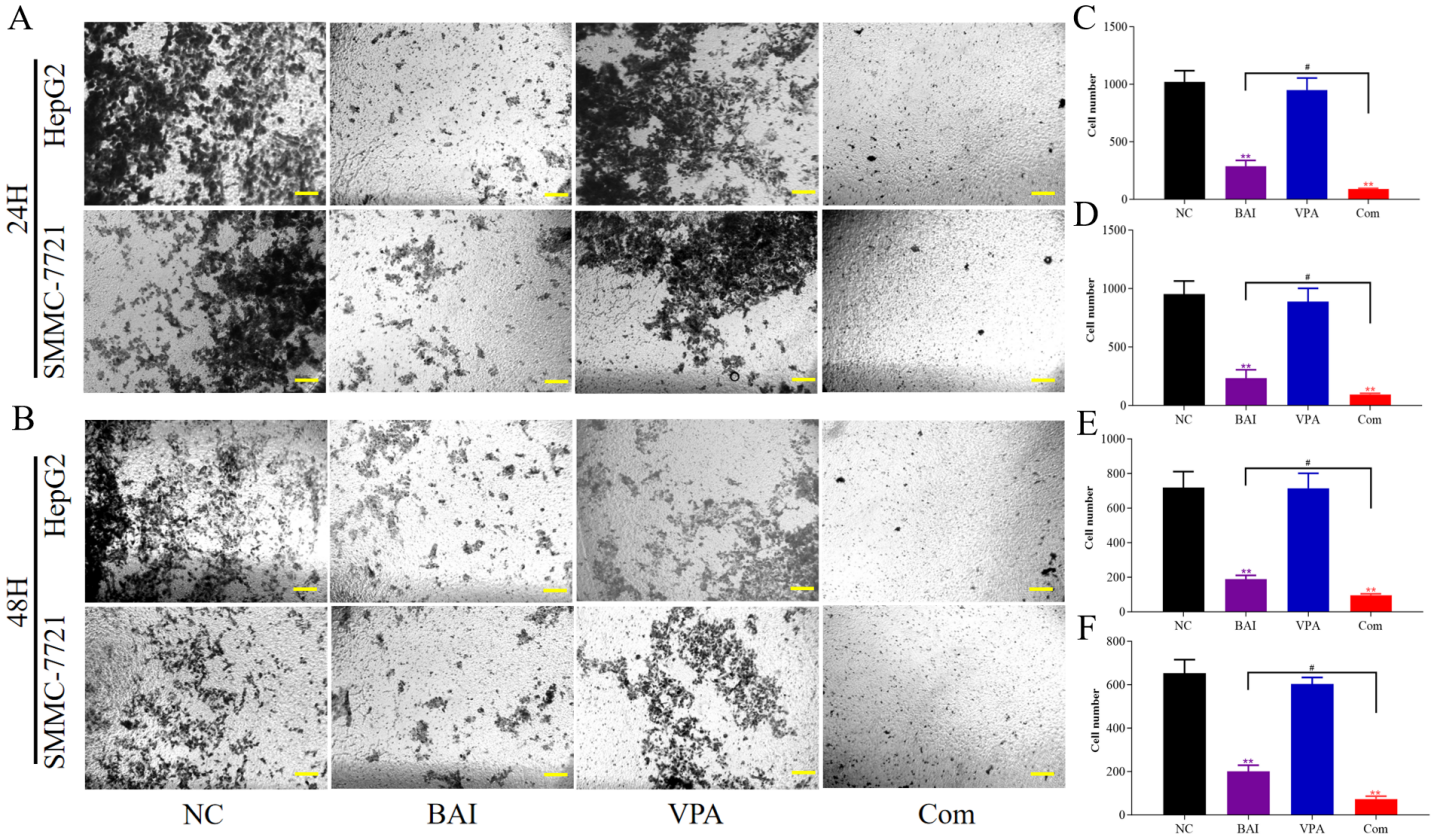
BAI and VPA inhibit the migratory ability of liver cancer cells. **(A, B)** Representative images of the transwell migration assay. HepG2 **(A)** and SMMC-7721 **(B)** cells were treated with BAI (40 μM), VPA (0.5 mM), or their combination (Com), and the migrated cells were stained and photographed after 24 h and 48 h of incubation. Scale bar, 50 μm. **(C-F)** Quantitative analysis of the number of migrated cells. The relative number of migrated cells was counted for HepG2 cells at 24 h **(C)** and 48 h **(D)**, and for SMMC-7721 cells at 24 h **(E)** and 48 h **(F)**. NC, Negative control; BAI, Baicalin; VPA, Valproic acid; Com, BAI+VPA. *compared with the NC group; *p < 0.05; **p < 0.01; ^#^compared with the Com group; ^#^p < 0.05.

**Figure 4:**
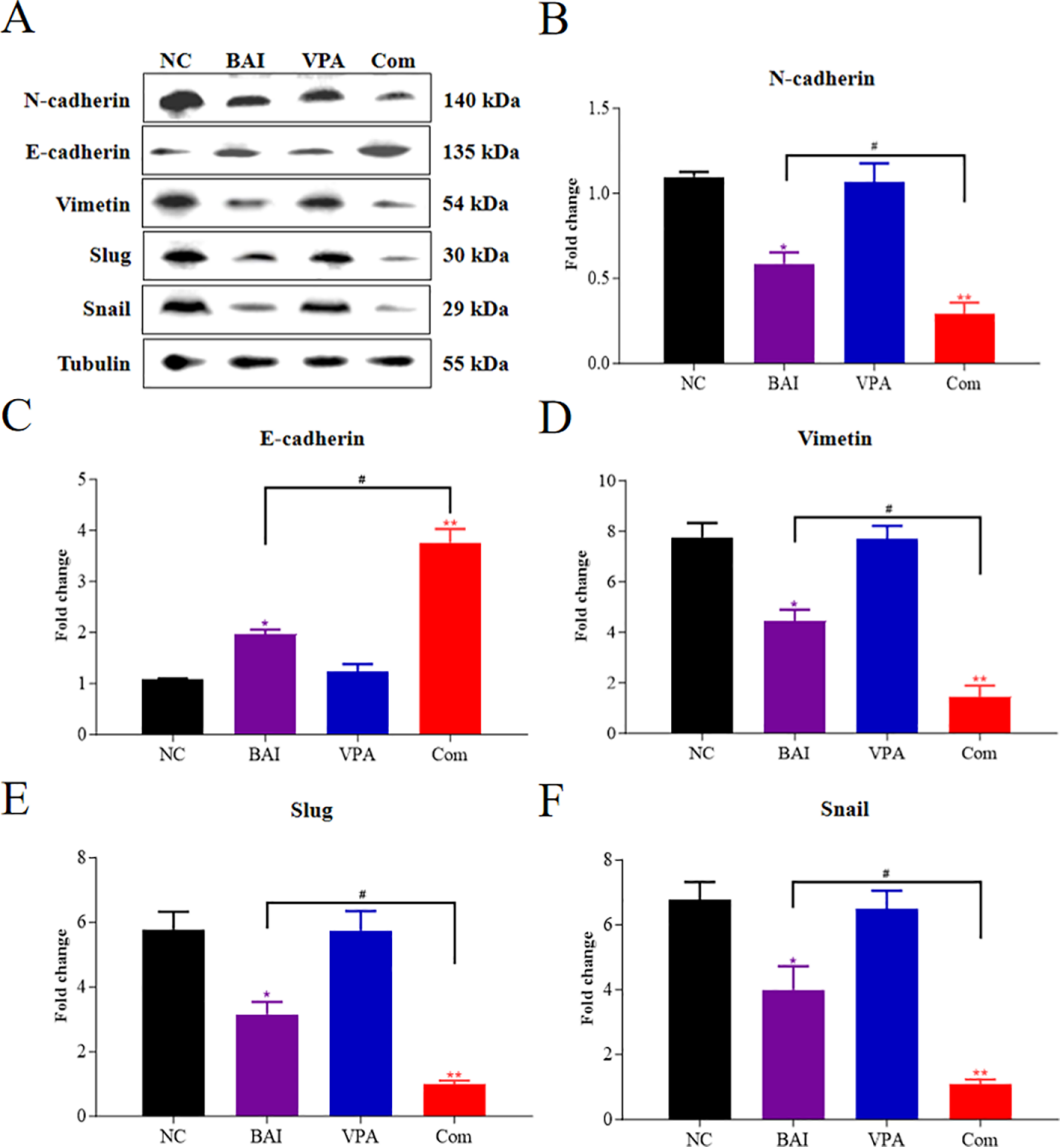
BAI and VPA regulate the expression of epithelial-mesenchymal transition (EMT)-related proteins in liver cancer cells. **(A)** Representative western blot images showing the protein levels of N-cadherin, E-cadherin, vimentin, slug, and snail in liver cancer cells treated with BAI (40 μM), VPA (0.5 mM), or their combination (Com). Tubulin served as the loading control. **(B–F)** Quantitative analysis of the relative protein expression levels of N-cadherin **(B)**, E-cadherin **(C)**, vimentin **(D)**, slug **(E)**, and snail **(F)**. NC, Negative control; BAI, Baicalin; VPA, Valproic acid; Com, BAI+VPA. *compared with the NC group; *p < 0.05; **p < 0.01; ^#^compared with the Com group; ^#^p < 0.05.

When liver cancer patients develop metastasis, treatment becomes considerably challenging, as tumor dissemination heavily depends on the migratory capacity of cancer cells. Consequently, suppressing the migration of tumor cells is essential for mitigating the detrimental impacts of metastasis. In this study, wound healing and transwell migration assays were employed to assess cellular migratory activity. The results demonstrated that BAI effectively reduced the wound closure rate of HepG2 and SMMC-7721 cells at the wound site, with the wound closure rate decreased by approximately 30-40% compared with the NC group at 48 h. In contrast, cell migration in the VPA group was largely unaffected, with wound closure rates remaining comparable to the NC group; however, VPA significantly augmented the inhibitory effect of BAI on the migration of liver cancer cells, reducing the wound closure rate by an additional ~20% in the Com group relative to the BAI monotherapy (**Figure 2**). The findings from the transwell migration assay further corroborated this observation. Compared with the NC group, the number of cells traversing the polycarbonate membrane was markedly decreased in both the BAI and Com groups: the BAI group showed a ~70% reduction in migrated cells, while the Com group exhibited the lowest number of migrated cells, with a reduction of more than 90% relative to the NC group. In contrast, no significant difference was observed between the VPA group and the NC group (**Figure 3**). Furthermore, western blot analysis was conducted to evaluate the expression levels of migration-related proteins in HepG2 cells. The results showed that, compared with the NC group, the expression levels of mesenchymal markers (N-cadherin, vimentin, snail, and slug) were significantly downregulated in the BAI group and Com group, with the most obvious downregulation observed in the Com group. Specifically, N-cadherin expression was reduced to ~0.6-fold in the BAI group and ~0.25-fold in the Com group; vimentin expression decreased to ~0.5-fold in the BAI group and ~0.2-fold in the Com group; snail and slug levels were reduced to ~0.5-fold in the BAI group and ~0.1-0.2-fold in the Com group. On the contrary, the expression level of the epithelial marker E-cadherin was upregulated in both the BAI group and Com group, with the Com group exhibiting the highest expression level, showing a ~2-fold increase in the BAI group and ~3.8-fold increase in the Com group relative to the NC group. Notably, protein expression in the VPA group showed no significant changes compared to the NC group (**Figure 4**).

### 3.3 VPA enhances the potential of BAI to induce apoptosis in liver cancer cells

**Figure 5:**
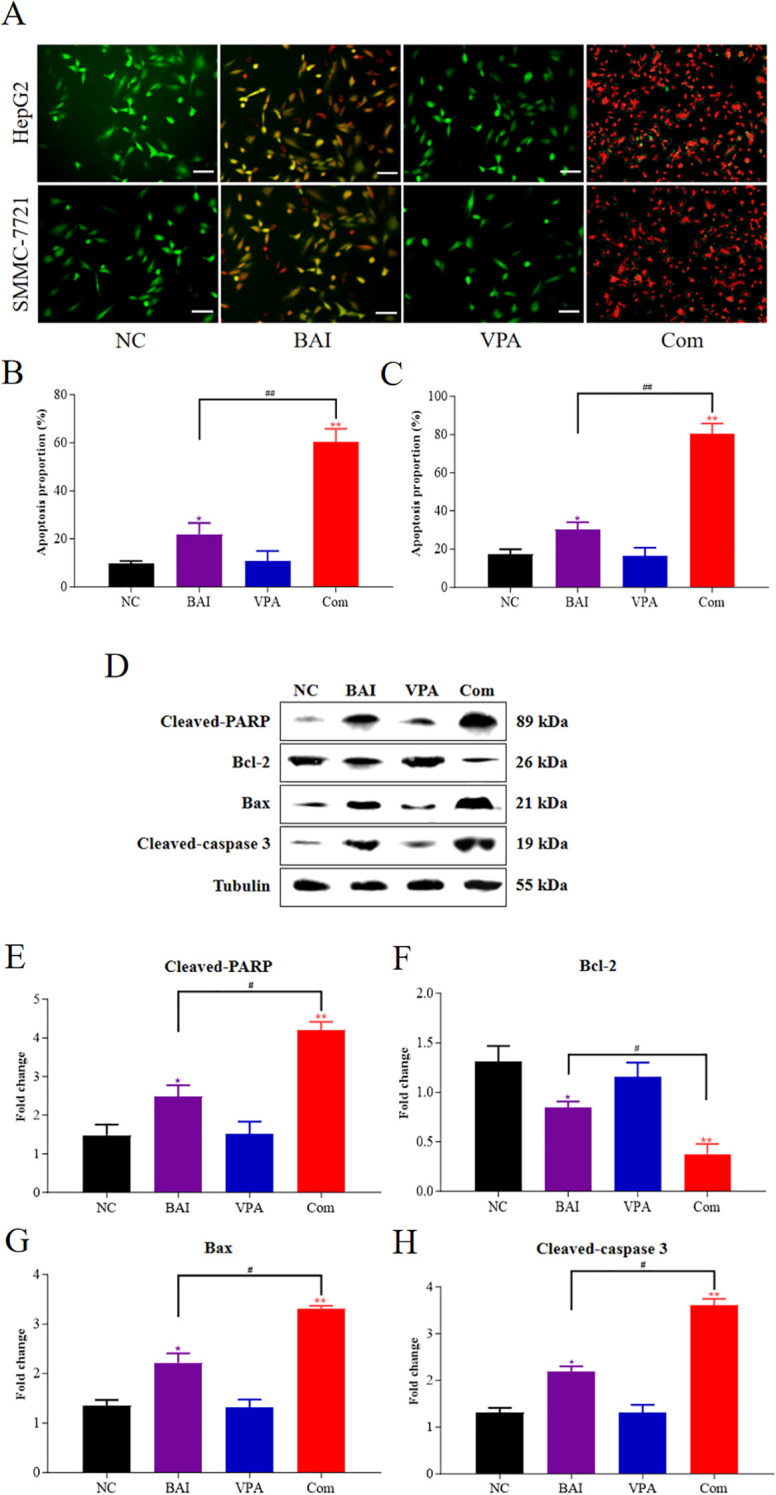
BAI and VPA synergistically induce apoptosis in liver cancer cells. **(A)** Representative fluorescence images of HepG2 and SMMC-7721 cells stained with an apoptosis detection kit, following treatment with BAI (40 μM), VPA (0.5 mM), or their combination (Com) for 24 h. Scale bar: 50 μμm. **(B, C)** Quantitative analysis of the apoptotic rate in HepG2 **(B)** and SMMC-7721 **(C)** cells. **(D)** Representative western blot images showing the expression levels of apoptosis-related proteins (cleaved-PARP, Bcl-2, Bax, cleaved-caspase 3). Tubulin served as the loading control. **(E–H)** Quantitative analysis of the relative protein expression levels of cleaved-PARP **(E)**, Bcl-2 **(F)**, Bax **(G)**, and cleaved-caspase 3 **(H)**. NC, Negative control; BAI, Baicalin; VPA, Valproic acid; Com, BAI+VPA. *compared with the NC group; *p < 0.05; **p < 0.01; ^#^compared with the Com group; ^#^p < 0.05; ^##^p < 0.01.

The apoptosis status was assessed using the AO/EB dual fluorescence staining method. Under fluorescence microscopy, viable cells exhibited green fluorescence due to AO uptake, whereas apoptotic cells displayed orange-red fluorescence as a result of EB uptake. Compared with the NC group, the number of cells exhibiting orange-red fluorescence was significantly increased in both the BAI group and the Com group. Quantitatively, the apoptotic rate in the BAI group reached approximately 20-30%, while the Com group showed the highest apoptotic proportion, with rates exceeding 60% in both HepG2 and SMMC-7721 cells. Notably, no significant difference in the number of red-fluorescent cells was observed between the NC group and the VPA group (**Figures 5A-C**). These findings indicate that BAI induces apoptosis in liver cancer cells, and at the concentration used in this study-where VPA alone exerts no significant effect-VPA potentiates the pro-apoptotic activity of BAI.

Western blot analysis was employed to assess the expression levels of apoptosis-related proteins in HepG2 cells. Compared with the NC group, the BAI group exhibited a significant upregulation in the expression of cleaved-caspase-3, Bax, and cleaved-PARP, and a marked downregulation of Bcl-2. Specifically: cleaved-PARP expression increased to ~2.5-fold in the BAI group and ~4-fold in the Com group; Bax expression increased to ~2.2-fold in the BAI group and ~3.3-fold in the Com group; cleaved-caspase-3 expression increased to ~2.2-fold in the BAI group and ~3.5-fold in the Com group; and Bcl-2 expression was reduced to ~0.8-fold in the BAI group and ~0.4-fold in the Com group. Furthermore, the expression pattern of these apoptosis-related proteins in the Com group closely resembled that observed in the BAI group, but the effects were significantly more pronounced (**Figures 5D–H**). These findings indicate that BAI induces substantial alterations in the expression of key regulatory proteins involved in apoptosis. When compared with the BAI group, the combination treatment in the Com group exerted a similar yet stronger effect on the expression of these apoptotic markers. Collectively, the experimental results support the conclusion that VPA enhances BAI-induced apoptosis in liver cancer cells.

### 3.4 BAI-VPA combination therapy exhibits anti-tumor effects on liver cancer cells comparable to DOX

**Figure 6:**
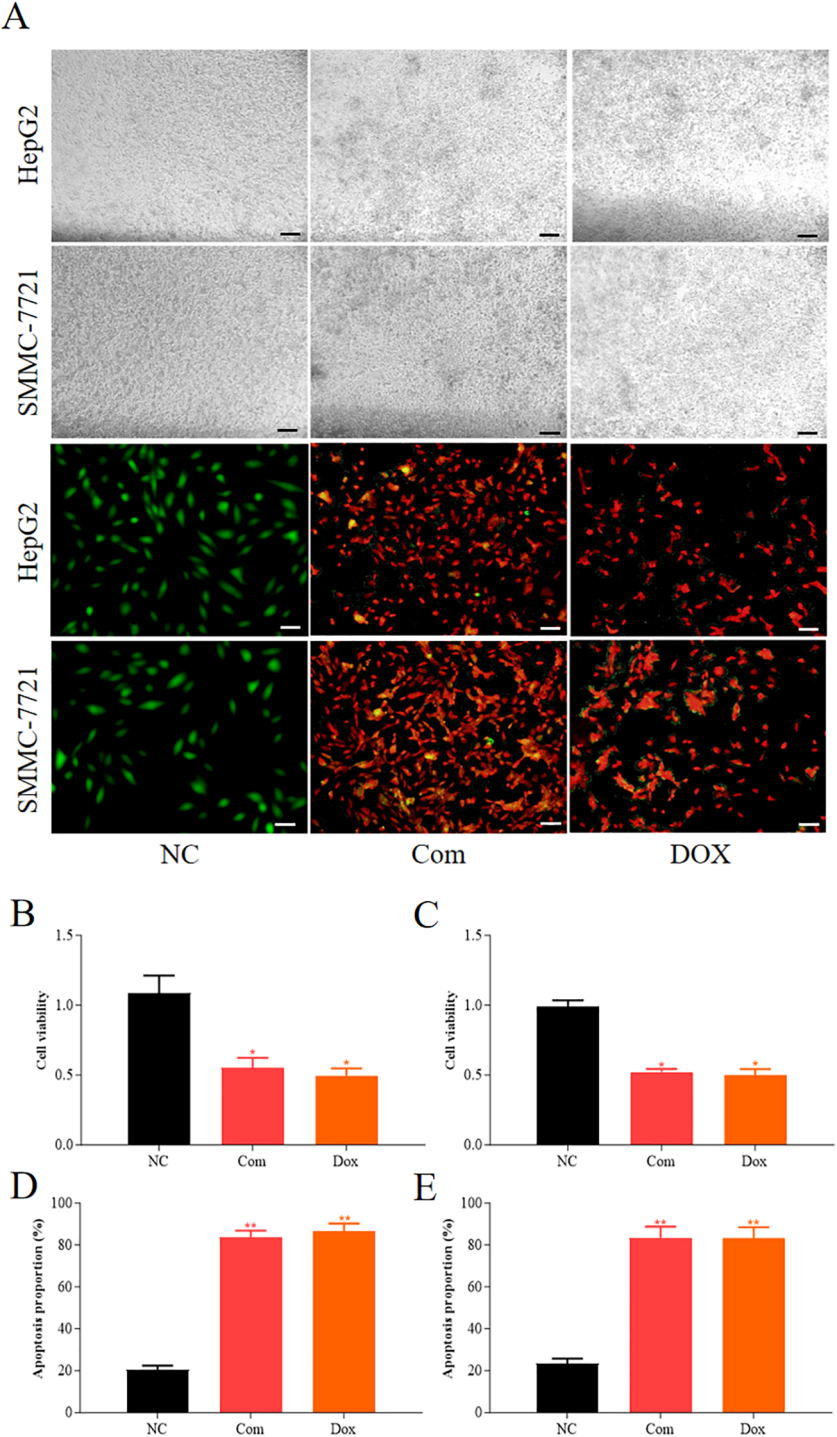
The BAI-VPA combination exhibits comparable anti-tumor efficacy to DOX in liver cancer cells. **(A)** Representative phase-contrast images (upper panels) showing morphological changes in HepG2 and SMMC-7721 cells after 24 h treatment with Com (40 μM BAI and 0.5 mM VPA) or DOX (0.5 μM). AO/EB fluorescence staining images (lower panels) visualize apoptotic cell death under the same conditions. Scale bar, 100 μm. **(B, C)** Quantitative analysis of cell viability in HepG2 **(B)** and SMMC-7721 **(C)** cells. **(D, E)** Quantitative analysis of the apoptotic proportion in HepG2 **(D)** and SMMC-7721 **(E)** cells. NC, Negative control; BAI, Baicalin; VPA, Valproic acid; Com, BAI+VPA; DOX, Doxorubicin. *compared with the NC group; *p < 0.05; **p < 0.01.

We assessed the cell growth of HepG2 and SMMC-7721 cells following incubation with the combination (Com, BAI+VPA) and DOX (0.5 μM) through morphological analysis. The results revealed that, in the NC group, both cell lines exhibited tight adherence and high cellular density. In contrast, the Com group and the DOX group displayed a marked reduction in cell density and significantly weakened adhesion, with scattered particles indicative of cellular damage observed within the field of view. Quantitative analysis showed that cell viability was reduced to approximately 50% in both the Com and DOX groups, representing a ~50% decrease relative to the NC group. No significant difference in proliferation inhibition was observed between the two treatments, suggesting that the growth-inhibitory effect of the BAI and VPA combination on liver cancer cells is comparable to that of DOX (**Figures 6A–C**). Similar observations were obtained in the cell apoptosis assay. Dual fluorescence staining with AO/EB showed that cells in the NC group emitted predominantly green fluorescence, indicative of viable cells. In contrast, the Com group and the DOX group exhibited a significant increase in orange-red fluorescence, representing apoptotic cells, with comparable intensities of apoptotic signals in both cell types. Further quantitative evaluation confirmed that the apoptotic proportion in the NC group was approximately 20%, while both the Com and DOX groups showed apoptotic rates exceeding 80%, representing a more than 4-fold increase in apoptosis compared with the NC group. No significant difference in the proportion of apoptotic cells was observed between the Com and DOX groups, indicating that the pro-apoptotic efficacy of the BAI and VPA combination is consistent with that of DOX (**Figures 6A, D, E**). These findings collectively demonstrate that the combined use of BAI and VPA effectively suppresses the proliferation of liver cancer cells and induces apoptosis, with potency comparable to that of DOX, a drug commonly used in clinical practice.

### 3.5 BAI-VPA combination-treatment augments ROS accumulation in liver cancer cells

**Figure 7:**
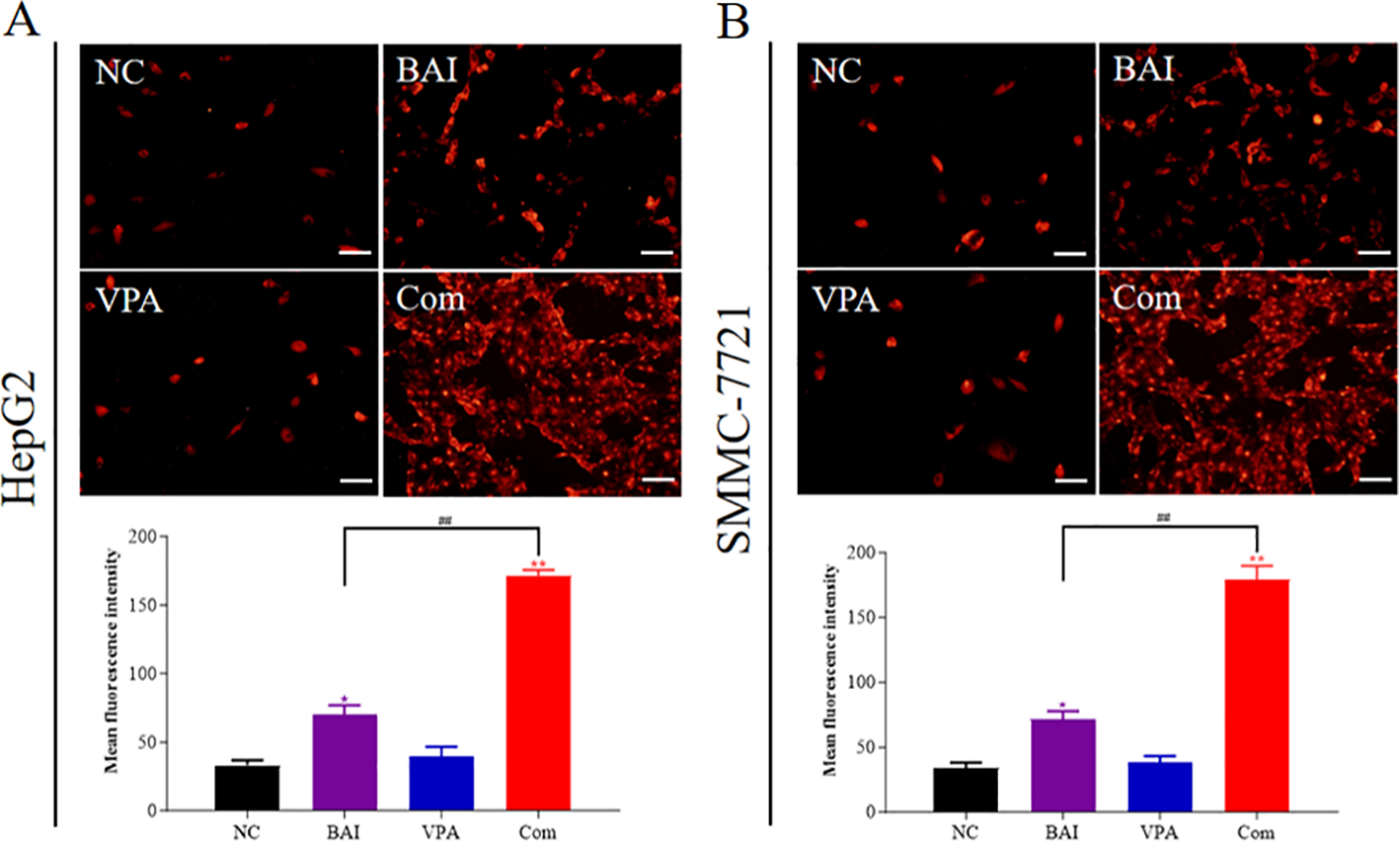
BAI and VPA synergistically promote intracellular ROS accumulation in liver cancer cells. **(A, B)** Representative fluorescence images showing ROS accumulation in HepG2 **(A)** and SMMC-7721 **(B)** cells treated with BAI (40 μM), VPA (0.5 mM), or their combination (Com) for 6 h. Scale bar, 50 μm. The lower panels display the quantitative analysis of mean fluorescence intensity for each group. NC, Negative control; BAI, Baicalin; VPA, Valproic acid; Com, BAI+VPA. *compared with the NC group; *p < 0.05; **p < 0.01; ^#^compared with the Com group; ^#^p < 0.05; ^##^p < 0.01.

In this study, DHE was employed as a fluorescent probe to assess ROS levels in liver cancer cells, with fluorescence intensity quantified using fluorescence microscopy. Among the four experimental groups, the Com group exhibited the highest fluorescence intensity, indicating the most pronounced ROS accumulation. The BAI group showed higher fluorescence intensity than the NC group, though lower than that of the Com group. In contrast, no significant difference in fluorescence intensity was observed between the NC and VPA groups (**Figures 7A, B**). Previous studies have demonstrated that BAI can promote intracellular ROS accumulation to exert its anti-tumor effects (24). To investigate whether the combination of BAI and VPA could synergistically amplify this effect, we used the fluorescent probe DHE to detect ROS levels in HepG2 and SMMC-7721 liver cancer cells, with fluorescence intensity quantified by fluorescence microscopy. In HepG2 cells (**Figure 7A**), the NC group showed minimal basal ROS fluorescence, while the BAI group exhibited a moderate increase in red fluorescence intensity, with ROS levels increased by approximately 2-fold compared to the NC group, indicating a significant induction of ROS accumulation. The VPA group, however, showed no significant difference in fluorescence intensity from the NC group, suggesting that VPA alone does not induce substantial ROS accumulation. Notably, the Com group displayed the most intense red fluorescence, with ROS levels reaching more than 4-fold those of the NC group and approximately 2.5-fold those of the BAI group, confirming a synergistic effect of BAI and VPA in promoting ROS production. Consistent results were observed in SMMC-7721 cells (**Figure 7B**). The NC group showed low baseline ROS levels, and the VPA group did not differ significantly from the NC group. The BAI group showed a notable increase in fluorescence intensity, with ROS levels approximately 2-fold higher than the NC group, while the Com group exhibited the highest ROS fluorescence intensity, reaching more than 4-fold the level in the NC group and approximately 2.5-fold that of the BAI group. These results confirm that BAI and VPA act synergistically to promote ROS accumulation in liver cancer cells, with BAI serving as the primary inducer of ROS and VPA enhancing this effect when combined.

### 3.6 Reduction of ROS levels abrogates the synergistic cytotoxic effect of BAI and VPA on liver cancer cells

**Figure 8:**
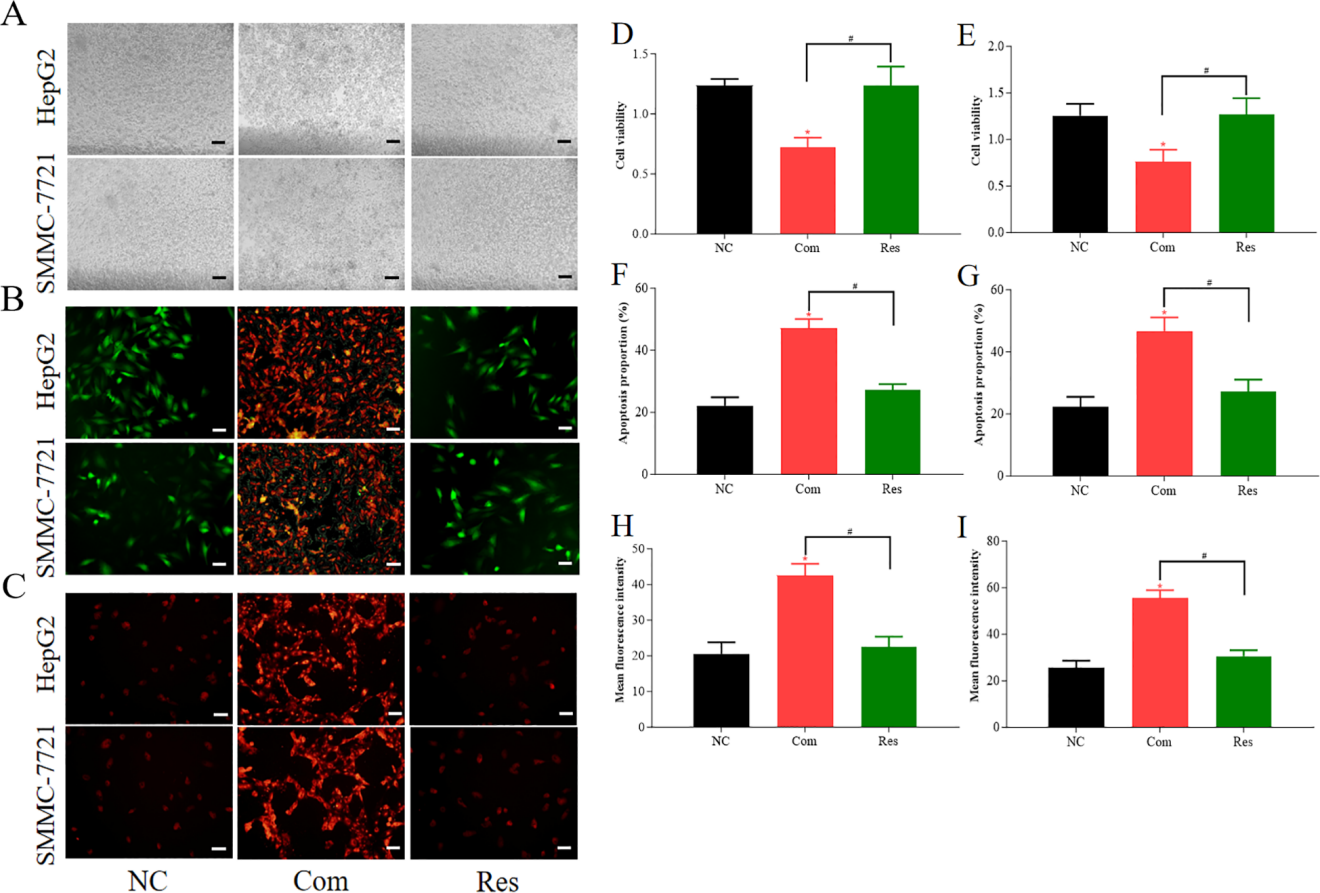
NAC reverses the anti-tumor effects of the BAI-VPA combination (Com) in liver cancer cells. **(A)** Representative phase-contrast images showing morphological alterations in HepG2 and SMMC-7721 cells after 24 h treatment with NC, Com, or Com+NAC (5 mM). Scale bar, 100 μm. **(B)** AO/EB fluorescence staining images visualizing apoptotic cell death in the corresponding groups (24 h post-treatment). Scale bar, 50 μm. **(C)** DHE-DA staining images assessing intracellular ROS accumulation in each group (6 h post-treatment). **(D, E)** Quantitative analysis of cell viability in HepG2 **(D)** and SMMC-7721 **(E)** cells. **(F, G)** Quantitative analysis of the apoptotic proportion in HepG2 **(F)** and SMMC-7721 **(G)** cells. **(H, I)** Quantitative analysis of mean fluorescence intensity (reflecting ROS levels) in HepG2 **(H)** and SMMC-7721 **(I)** cells. NC, Negative control; BAI, Baicalin; VPA, Valproic acid; Com, BAI+VPA; NAC, N-acetylcysteine. *compared with the NC group; *p < 0.05; **p < 0.01; ^#^compared with the Com group; ^#^p < 0.05; ^##^p < 0.01.

To determine whether ROS accumulation plays a critical role in inducing apoptosis in liver cancer cells, we employed NAC (5 mM), a well-established antioxidant (25), to attenuate intracellular ROS levels. First, we repeated the ROS detection assay to validate the efficacy of NAC in reducing ROS. In the DHE staining experiments, treatment with BAI and VPA in combination markedly enhanced ROS accumulation in both HepG2 and SMMC-7721 cells (**Figure 8C**). Quantitative analysis of DHE fluorescence intensity further confirmed that the Com group exhibited significantly higher ROS levels than the NC group, with mean fluorescence intensity increased by approximately 2-fold compared with baseline (**Figures 8H, I**). Notably, the fluorescence intensity in the Res group-treated with NAC alongside BAI and VPA-was substantially lower compared to the Com group, showing a more than 50% reduction, and nearly returned to the baseline levels of the NC group (**Figures 8H, I**), confirming that NAC effectively reduces ROS levels in liver cancer cells.

We then captured phase-contrast microscopic images of HepG2 and SMMC-7721 cells treated with BAI and VPA, both in the presence and absence of NAC, after 24 h of incubation. Microscopic observation showed that cells in the NC group maintained high density with intact intercellular connections. In contrast, cells in the Com group exhibited obvious morphological damage, including reduced cell adhesion, detachment from the culture surface, and the appearance of granular substances, all of which are typical characteristics of cellular apoptosis and cytotoxic stress. Notably, cells in the Res group regained favorable adhesion capacity, and the cell number was comparable to that of the NC group (**Figure 8A**). These morphological changes were further supported by quantitative CCK-8 assay, which demonstrated that the Com group exhibited a significant reduction in cell viability compared to the NC group, with viability dropping to approximately 60-70% of control levels. NAC co-treatment in the Res group significantly restored cell proliferation to near-control levels, reaching more than 90% of the NC group viability (**Figures 8D, E**). These findings collectively demonstrate that attenuation of ROS levels can reverse the cytotoxic effects induced by the combination of BAI and VPA in liver cancer cells.

The accumulation of ROS has been shown to induce cell apoptosis (26). To further investigate whether the induction of apoptosis is associated with ROS accumulation resulting from treatment with BAI and VPA, we employed NAC as an antioxidant agent and evaluated apoptosis using AO/EB dual fluorescence staining. AO/EB staining allows for the clear distinction between live cells (green fluorescence), early apoptotic cells (green-yellow fluorescence), and late apoptotic/necrotic cells (orange-red fluorescence). Compared with the NC group, which showed minimal apoptotic cells (~20%), the Com group exhibited a marked increase in the number of early and late apoptotic cells, with the apoptotic rate rising to approximately 45-50%, consistent with our previous findings (**Figure 8B**). Quantitative analysis of apoptotic rates confirmed that the Com group had a significantly higher apoptosis rate than the NC group (**Figures 8F, G**). Notably, upon co-treatment with NAC, the apoptosis rate in the Res group was significantly reduced compared to the Com group, dropping to approximately 25-30%, and was nearly restored to the level observed in the NC group (**Figures 8B, F, G**). These results suggest that BAI- and VPA-induced ROS accumulation plays a critical role in triggering apoptosis in liver cancer cells.

### 3.7 BAI and VPA synergistically induce cell apoptosis through the ROS-PTEN-Bax axis

**Figure 9:**
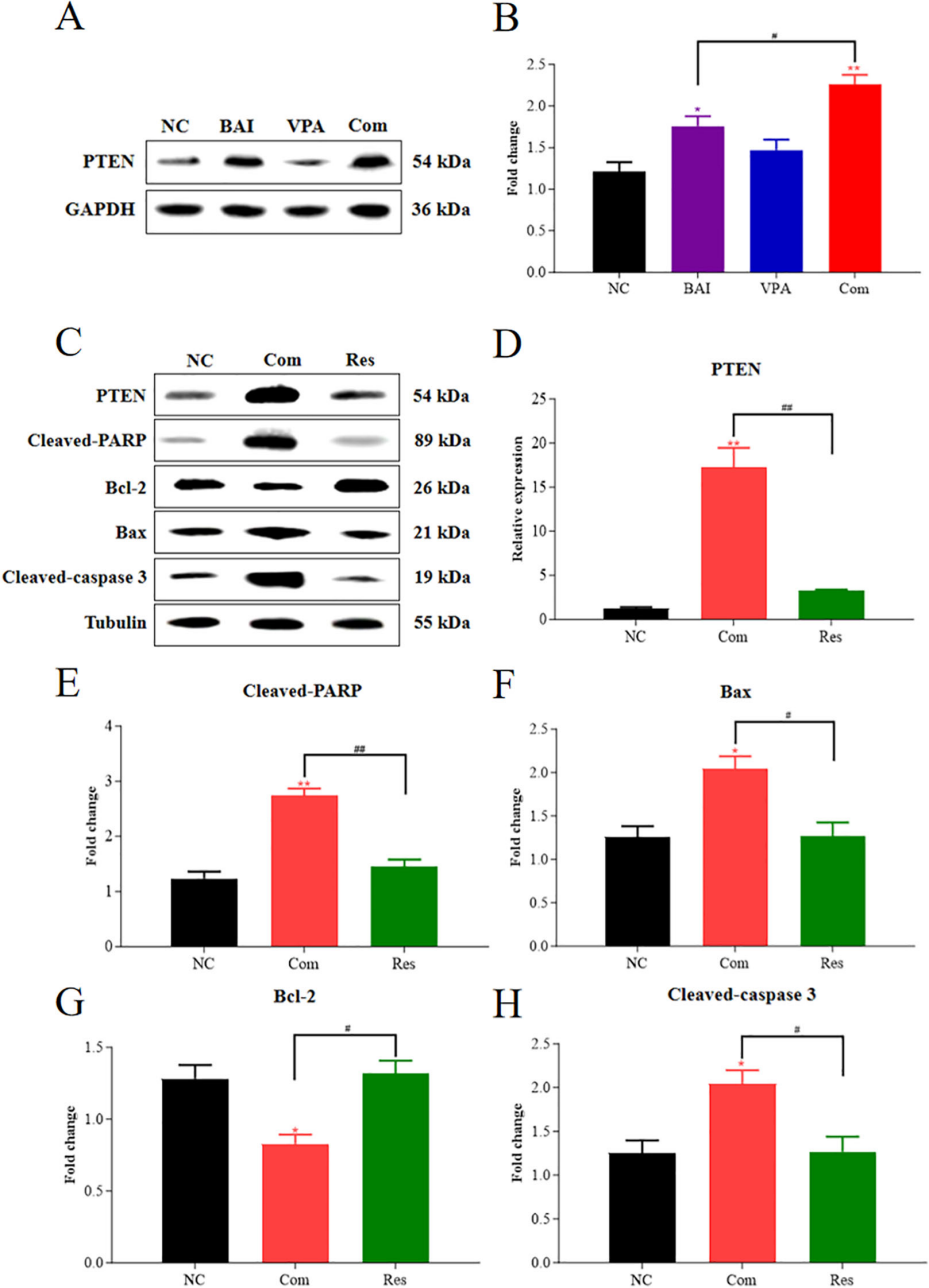
NAC reverses the BAI-VPA combination (Com)-induced upregulation of PTEN and apoptosis in liver cancer cells. **(A)** Representative western blot images showing PTEN protein expression in cells treated with NC, BAI, VPA, or Com for 24 h. GAPDH served as the loading control. **(B)** Quantitative analysis of relative PTEN protein expression levels. **(C)** Representative western blot images showing the expression of PTEN and apoptosis-related proteins (cleaved-PARP, Bax, Bcl-2, cleaved-caspase 3) in cells treated with NC, Com, or Com+NAC (labeled as Res) for 24 h. Tubulin served as the loading control. **(D–H)** Quantitative analysis of the relative protein expression levels of PTEN **(D)**, cleaved-PARP **(E)**, Bax **(F)**, Bcl-2 **(G)**, and cleaved-caspase 3 **(H)**. NC, Negative control; BAI, Baicalin; VPA, Valproic acid; Com, BAI+VPA; NAC (Res), N-acetylcysteine (labeled as Res in the figure). *compared with the NC group; *p < 0.05; **p < 0.01; ^#^compared with the Com group; ^#^p < 0.05; ^##^p < 0.01.

To further explore the mechanistic connection between ROS accumulation and apoptosis, we focused on the key regulatory gene PTEN, which has been proposed to act as a molecular bridge between ROS accumulation and apoptotic pathways (26). Western blot analysis confirmed that BAI treatment significantly upregulated PTEN protein expression in HepG2 cells, with the highest expression observed in the Com group, whereas no significant difference was detected between the VPA and NC groups (**Figure 9A**). Quantitative analysis of PTEN protein levels further validated these observations, showing a 2.2-fold increase in the Com group compared to the NC group (**Figure 9B**). To verify whether the BAI-VPA combination induces apoptosis through the ROS-PTEN-Bax signaling axis, we pretreated HepG2 cells with the ROS scavenger NAC and examined PTEN expression via western blot analysis. The results revealed that BAI-VPA treatment significantly increased PTEN protein expression, and this upregulation was strongly inhibited by ROS scavenging with NAC (**Figures 9C, D**). This finding directly links ROS accumulation to PTEN upregulation, confirming that ROS is an upstream regulator of PTEN expression in this context.

Consistently, the expression of apoptosis-related proteins exhibited a parallel pattern to PTEN expression. Specifically, elevated PTEN expression was associated with increased Bax levels, reduced Bcl-2 expression, and higher levels of cleaved-caspase 3 and cleaved-PARP (**Figures 9C, E–H**). Quantitative densitometric analysis confirmed that the Com group exhibited a 2.5-fold increase in Bax protein levels, a 40% reduction in Bcl-2 levels, and a 3-fold increase in cleaved-caspase 3 and cleaved-PARP levels compared to the NC group. In contrast, NAC co-treatment in the Res group significantly attenuated these changes, restoring the expression of these apoptotic markers to near-control levels (**Figures 9E-H**). These results confirm that the synergistic induction of apoptosis by BAI and VPA is mediated via the ROS-PTEN-Bax axis.

**Figure 10:**
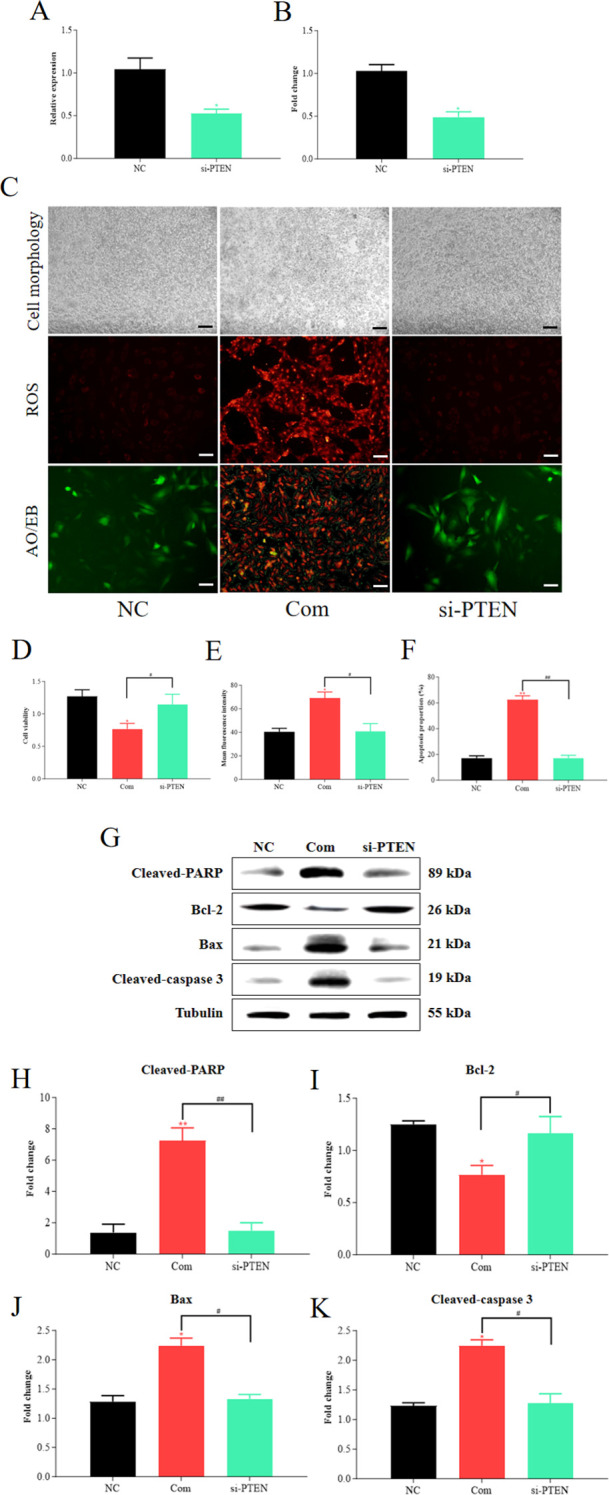
PTEN knockdown reverses the anti-tumor effects of the BAI-VPA combination (Com) in liver cancer cells. **(A, B)** Verification of PTEN knockdown efficiency in liver cancer cells using siRNA, assessed by quantitative PCR **(A)** and western blot **(B, C)** Representative images of cell morphology (phase-contrast), intracellular ROS accumulation (DHE-DA staining), and apoptotic cell death (AO/EB staining) in NC, Com-treated, and si-PTEN-transfected cells (24 h post-treatment). Scale bar, 100 μm (cell morphology); 50 μm (ROS, AO/EB). **(D)** Quantitative analysis of cell viability in each group. **(E)** Quantitative analysis of mean fluorescence intensity reflecting ROS levels. **(F)** Quantitative analysis of the apoptotic proportion in each group. **(G)** Representative western blot images showing the expression of apoptosis-related proteins (cleaved-PARP, Bcl-2, Bax, cleaved-caspase 3) in NC, Com-treated, and si-PTEN-transfected cells. Tubulin served as the loading control. **(H-K)** Quantitative analysis of the relative protein expression levels of cleaved-PARP **(H)**, Bcl-2 **(I)**, Bax **(J)**, and cleaved-caspase 3 **(K)**. NC, Negative control; BAI, Baicalin; VPA, Valproic acid; Com, BAI+VPA. *compared with the NC group; *p < 0.05; **p < 0.01; ^#^compared with the Com group; ^#^p < 0.05; ^##^p < 0.01.

To further confirm that the BAI-VPA combination induces apoptosis through the PTEN-Bax pathway, we generated PTEN-knockdown HepG2 cells using siRNA. Quantitative PCR and western blot analysis confirmed that PTEN expression was efficiently knocked down at both the mRNA and protein levels, with expression reduced to approximately 50% of the control level (**Figures 10A, B**). Functional assays demonstrated that PTEN knockdown significantly increased the resistance of HepG2 cells to BAI-VPA combination treatment. Specifically, cell proliferation assays (**Figure 10D**) showed that the Com group caused a marked reduction in cell viability to approximately 60% of the NC group, while PTEN-knockdown cells exhibited a significant recovery in proliferation capacity, reaching more than 90% of control viability, confirming that PTEN deficiency attenuates the cytotoxic effects of the BAI-VPA combination. Morphological analysis showed that, compared to control cells, PTEN-knockdown cells maintained stronger adhesion and higher cell density after combined treatment (**Figure 10C**). AO/EB staining further confirmed that PTEN knockdown reduced the number of apoptotic cells induced by the BAI-VPA combination (**Figures 10C, F**): the apoptotic rate in the Com group reached approximately 60%, while in the si-PTEN group it dropped to around 20%, similar to baseline levels. Western blot assays (**Figures 10G–K**) consistently demonstrated a reduced apoptotic response in PTEN-knockdown cells, as evidenced by decreased Bax expression, increased Bcl-2 levels, and reduced cleaved-caspase 3 and cleaved-PARP. Compared with the NC group, the Com group showed a 2.2-fold increase in Bax, a 35% reduction in Bcl-2, a 7-fold increase in cleaved-PARP, and a 2.2-fold increase in cleaved-caspase 3. In contrast, the si-PTEN group showed only a 1.3-fold increase in Bax, near-baseline Bcl-2 levels, a 1.5-fold increase in cleaved-PARP, and a 1.3-fold increase in cleaved-caspase 3 compared with controls. Notably, the fluorescence intensity of the ROS probe in the si-PTEN group was significantly weaker than that in the Com group (**Figures 10C, E**): the Com group showed a nearly 2-fold increase in ROS levels compared with NC, while the si-PTEN group had ROS levels close to baseline, indicating that PTEN is not only a downstream target of ROS but also involved in regulating ROS accumulation, thus forming a positive feedback loop. Collectively, these findings support the existence of a positive feedback loop between PTEN and ROS in liver cancer cells, which amplifies the apoptotic response induced by the BAI-VPA combination.

### 3.8 Inhibition of liver cancer growth by BAI and VPA combination treatment

**Figure 11:**
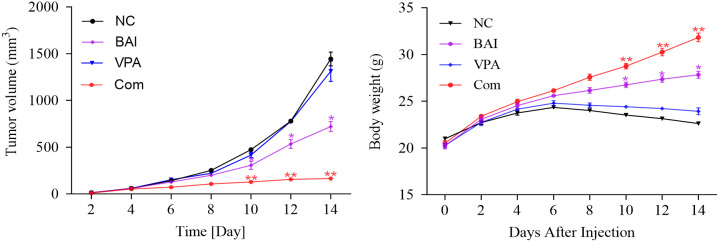
Results of *in vivo* experiments. **(A)** Tumor volume growth curve; **(B)** Mouse body weight change curve. NC, Negative control; BAI, Baicalin; VPA, Valproic acid; Com, BAI+VPA. *compared with the NC group; *p < 0.05; **p < 0.01.

*In vitro* experiments have demonstrated that the combination of BAI and VPA exerts a significant inhibitory effect on liver cancer cells. To further evaluate the *in vivo* antitumor efficacy of this combination, a subcutaneous syngeneic tumor model was established, followed by consecutive intraperitoneal injections for two weeks prior to sacrifice. Tumor volume and body weight were dynamically monitored throughout the study. As shown in **Figure 11A**, the combined treatment group exhibited a markedly superior tumor suppression effect compared to the BAI group, the VPA group, and the NC group. At day 14, the average tumor volume in the NC group reached approximately 1450 mm3, while the VPA group showed no significant difference, reaching around 1300 mm3. The BAI monotherapy group reduced tumor volume to about 700 mm3, representing a ~50% reduction relative to the NC group. Notably, the Com group achieved the strongest suppression, with a final tumor volume of only ~150 mm3, corresponding to an 89% reduction compared to the NC group and a 79% reduction compared to the BAI group alone. The tumor growth rate was the slowest in the Com group, with an average daily increase of less than 15 mm3 versus over 100 mm3 in the NC group.

Regarding body weight changes, the NC group showed distinctly lower body weight than all treatment groups, which could be explained by tumor burden-induced cachexia-like consumption. The NC group harbored the largest and fastest-growing tumor mass, triggering systemic metabolic disturbance and inflammatory activation, ultimately leading to nutrient consumption and restrained body weight gain. In contrast, all drug-treated groups, especially the Com group, obtained obvious tumor growth inhibition, which greatly alleviated tumor-derived systemic consumption. In addition, BAI has been reported to exert anti-inflammatory and metabolic protective effects, while VPA could indirectly reduce physiological consumption by suppressing tumor progression. The combined application further improved the general nutritional status of tumor-bearing mice, contributing to steady body weight growth.

Consistently, mice in the Com group maintained the most stable and favorable body weight gain, which was significantly higher than that observed in the NC group. By day 14, the average body weight in the Com group increased by more than 50% from baseline, reaching approximately 32 g, whereas the NC group showed only a slight weight gain of less than 10% and remained below 23 g. Moreover, the Com group outperformed the single-agent groups; although BAI and VPA monotherapy also partially restrained tumor growth, slight body weight fluctuations were still observed. The BAI group showed a moderate weight increase to approximately 28 g, while the body weight of the VPA group plateaued at around 24 g after day 6. These findings indicate that the combination of BAI and VPA not only enhances the inhibitory effect on liver cancer growth but also relieves tumor-induced systemic metabolic burden, thereby demonstrating dual advantages of synergistic anti-tumor activity and improved physiological tolerance *in vivo*.

## 4 Discussion

Liver cancer is one of the most prevalent and aggressive malignancies worldwide, characterized by a high incidence rate and poor prognosis. Its insidious nature, propensity for intrahepatic and distant metastasis, and tendency to develop drug resistance often prevent a substantial proportion of patients from achieving optimal therapeutic outcomes, even with systemic treatment (27). Furthermore, the adverse effects commonly associated with chemotherapy-such as nausea, vomiting, bone marrow suppression, and immunosuppression (28)-significantly impair patients’ quality of life. Therefore, there is an urgent need to identify effective therapeutic agents for the management of liver cancer. Although BAI has been demonstrated to exert anti-tumor effects against liver cancer (8), its clinical application is considerably limited by inherent toxicity and various pharmacological constraints. To overcome these limitations, this study repurposes VPA, a known histone deacetylase inhibitor, as an adjuvant therapy. The rationale is to enhance tumor cell sensitivity to BAI by promoting a more relaxed chromatin conformation, thereby facilitating gene expression and potentially improving therapeutic efficacy. This study represents the first investigation into the synergistic effects of combining BAI with VPA in the context of liver cancer. *In vitro* experimental results revealed that the combination of BAI and VPA significantly outperformed monotherapy with BAI in inhibiting liver cancer cell proliferation, demonstrating a clear synergistic interaction. BAI exerts cytotoxic effects on liver cancer cells through the induction of ROS accumulation. However, the precise molecular mechanisms by which ROS accumulation triggers apoptotic pathways remain complex and warrant further in-depth investigation. In this study, we confirmed through key molecular analyses, Western blotting, and a series of rigorous *in vitro* rescue experiments that the combined application of BAI and VPA induces apoptosis in liver cancer cells via the ROS-PTEN-Bax signaling axis. The results demonstrated that the addition of VPA significantly enhances the cytotoxic effect of BAI against liver cancer cells. Notably, even at relatively safe concentrations, VPA markedly potentiated this cytotoxicity.

For patients with liver cancer, the prognosis associated with metastasis-particularly liver metastasis-is often poor. Therefore, inhibiting cell migration is critical for preventing tumor dissemination. To evaluate the effects of BAI and VPA on the migratory capacity of liver cancer cells, we performed wound healing and transwell migration assays. Both the scratch wound healing and transwell migration results indicated that VPA enhances the anti-migratory efficacy of BAI. E-cadherin is a pivotal epithelial marker responsible for maintaining cell adhesion and tissue integrity, which can markedly inhibit the motility and migration of tumor cells. As the core transcriptional factors of EMT, snail1 and slug, along with mesenchymal markers such as vimentin, impair intercellular adhesive junctions and enhance cell motility. On the contrary, aberrant overexpression of N-cadherin in tumors mediates abnormal intercellular crosstalk, induces epithelial-mesenchymal transition, and ultimately facilitates the migration and distant metastasis of tumor cells. Western blot results demonstrated that the expression levels of N-cadherin, snail1, slug and vimentin were downregulated in the treatment groups, whereas E-cadherin expression was upregulated, with the most remarkable alterations observed in the Com group. These findings further substantiate the synergistic effectiveness of BAI and VPA in suppressing the migratory potential of liver cancer cells.

Inducing cell apoptosis plays a critical role in suppressing tumor progression. Results from the cell apoptosis staining assay demonstrated that the apoptotic rate in HepG2 cells was highest in the Com group, followed by the BAI group, with both groups exhibiting significantly higher apoptosis levels compared to the NC group and the VPA group. In SMMC-7721 cells, a similar trend was observed across the NC, BAI, VPA, and Com groups. The intrinsic apoptotic pathway is typically activated in response to endogenous stimuli such as irreparable DNA damage, hypoxia, elevated cytoplasmic Ca²^+^ concentrations, and severe oxidative stress. We further evaluated the expression levels of key apoptotic proteins-including Bax, Bcl-2, caspase-3, cleaved-caspase-3, and cleaved-PARP-using Western blot analysis. The results revealed that BAI treatment upregulated Bax expression while downregulating Bcl-2 expression. These alterations increased mitochondrial membrane permeability, facilitating the release of pro-apoptotic factors, which subsequently enhanced the activation of cleaved-caspase-3 and cleaved-PARP, ultimately promoting apoptotic cell death. Notably, VPA exhibited a synergistic enhancing effect in this process.

BAI exerts its anti-tumor effect by promoting the accumulation of ROS, a finding that has been confirmed by our study. To assess intracellular ROS levels across different experimental groups, we employed ROS-specific fluorescent probes. Given the inherently rapid degradation rate of ROS, we optimized the detection protocol by shortening the incubation time from 24 h to 6 h. The results demonstrated that the Com group exhibited the highest level of ROS accumulation, followed by the BAI group, with both groups showing significantly elevated ROS levels compared to the NC group and the VPA group. Notably, the observed trend in ROS accumulation paralleled the extent of cellular apoptosis, suggesting a close association between ROS buildup and the apoptotic process in liver cancer cells. Furthermore, our rescue experiments confirmed that ROS scavenging with NAC significantly abrogated the upregulation of PTEN induced by BAI-VPA combination treatment, directly validating ROS as an upstream regulator of PTEN. Activated PTEN subsequently modulates the expression of Bax, a key pro-apoptotic protein in the intrinsic mitochondrial pathway, thereby enhancing apoptotic signaling and ultimately triggering HCC cell death. Based on these findings, we propose a mechanistic model for the synergistic induction of apoptosis by BAI in combination with VPA: ROS accumulation drives the upregulation of PTEN, which in turn activates the intrinsic apoptotic pathway through regulating Bax expression, ultimately promoting apoptosis in HepG2 cells.

To verify our hypothesis, we performed a Western blot analysis to evaluate the expression level of PTEN, and the results provided preliminary support for this hypothesis. To further investigate the underlying mechanism, we conducted an NAC rescue experiment, in which NAC was used as a ROS scavenger to reduce ROS accumulation in liver cancer cells. Subsequently, ROS detection assays demonstrated that the ROS concentration in the Res group was significantly lower than that in the Com group, confirming the effective ROS-scavenging activity of NAC. The reduction in ROS levels reversed the cytotoxicity induced by the combined treatment of BAI and VPA, and the apoptosis rate in the Res group was markedly decreased compared to the Com group. As ROS levels diminished, PTEN expression also declined, leading to reduced Bax expression and subsequent alterations in downstream apoptosis-related proteins. To further substantiate that the synergistic effect of BAI and VPA induces cell apoptosis via the PTEN-Bax signaling pathway, we performed an additional rescue experiment involving PTEN silencing. The results revealed that downregulation of PTEN partially enhanced the resistance of HepG2 cells to the BAI and VPA combination treatment; however, this protective effect was incomplete. Therefore, we hypothesize that, in addition to PTEN, other factors may also be involved in mediating the regulatory link between ROS and apoptosis. Interestingly, the results of the rescue experiment revealed a positive feedback regulatory loop between ROS accumulation and the upregulation of PTEN. By employing VPA to induce chromatin relaxation and thereby expose DNA, this cellular state further enhanced the aforementioned positive feedback mechanism. We hypothesize that this may represent a key sensitization mechanism underlying the action of VPA. It is evident that the reverse validation of this mechanism through rescue experiments has further strengthened our hypothesis. This study represents the first attempt to explore the combined application of BAI and VPA in the treatment of liver cancer, confirming the potent anti-tumor efficacy of BAI, which aligns with previous findings. Compared to monotherapy with BAI, the combination of BAI and VPA demonstrated significantly enhanced anti-tumor activity. Collectively, the *in vivo* experimental results indicate that the co-administration of BAI and VPA can effectively suppress tumor growth. Therefore, this combinatorial therapeutic strategy holds promise as a novel approach for liver cancer treatment. Although this study presents meaningful findings, several limitations should be acknowledged. First, the precise upstream mechanisms driving ROS accumulation remain unclear and warrant further investigation to elucidate their molecular details. Second, in addition to PTEN, other signaling pathways may also be involved in regulating ROS-induced apoptosis and require comprehensive exploration. Finally, the specific molecular mechanisms underlying the anti-migratory effects observed in this study deserve in-depth analysis in future research.

## 5 Conclusion

In conclusion, VPA acts as an effective sensitizer for BAI, significantly enhancing the antitumor efficacy of BAI against HCC *in vitro* and *in vivo*. The combination of BAI and VPA exerts synergistic effects by inhibiting HCC cell proliferation, suppressing cell migration, and promoting cell apoptosis, with efficacy comparable to the chemotherapeutic drug doxorubicin. Mechanistically, this synergistic antitumor effect is mediated through the ROS-PTEN-Bax signaling axis: BAI induces intracellular ROS accumulation, which upregulates PTEN expression, and the two form a positive feedback loop to modulate the expression of apoptosis-related proteins, ultimately inducing HCC cell apoptosis. This study provides a novel, safe, and clinically translatable combinatorial therapeutic strategy for liver cancer, while establishing an experimental foundation for repurposing VPA as an adjuvant agent to overcome the limitations of BAI monotherapy.

## Data Availability

The original contributions presented in the study are included in the article/supplementary material. Further inquiries can be directed to the corresponding author.

## References

[1] Chaturvedi VK Singh A Dubey SK Hetta HF John J Singh MP . Molecular mechanistic insight of hepatitis B virus mediated hepatocellular carcinoma. Microb Pathog. (2019) 128:184–94. doi: 10.1016/j.micpath.2019.01.004. PMID: 30611768

[2] Marrocco GA Silletta M Bianco V Mondera F Sciortino C Pappalardo L . Lenvatinib versus sorafenib in advanced hepatic cell carcinoma: A double center retrospective analysis. Anticancer Res. (2023) 43:755–63. doi: 10.21873/anticanres.16215. PMID: 36697101

[3] Chidambaranathan-Reghupaty S Fisher PB Sarkar D . Hepatocellular carcinoma (HCC): Epidemiology, etiology and molecular classification. Adv Cancer Res. (2021) 149:1–61. doi: 10.1016/bs.acr.2020.10.001. PMID: 33579421 PMC8796122

[4] Wang MJ Chen F Gao JL Liu H Li X Hou J . Clusterin drives fiber endocytosis by mesothelial cells to resolve liver fibrosis. Gastroenterology. (2026) 170:569–83. doi: 10.1053/j.gastro.2025.08.022. PMID: 41369634

[5] Wang Y Wang Q Yang TW Yin JM Wei FL Liu H . Analysis of immune and inflammatory microenvironment characteristics of noncancer end-stage liver disease. J Interferon Cytokine Res. (2023) 43:86–97. doi: 10.1089/jir.2022.0172. PMID: 36749162

[6] Cui Y Liu J Wang X Wu Y Chang Y Hu X . Baicalin attenuates the immune escape of oral squamous cell carcinoma by reducing lactate accumulation in tumor microenvironment. J Adv Res. (2025) 77:721–32. doi: 10.1016/j.jare.2025.01.021. PMID: 39814222 PMC12627396

[7] Adeshakin AO Yan D Zhang M Wang L Adeshakin FO Liu W . Blockade of myeloid-derived suppressor cell function by valproic acid enhanced anti-PD-L1 tumor immunotherapy. Biochem Biophys Res Commun. (2020) 522:604–11. doi: 10.1016/j.bbrc.2019.11.155. PMID: 31785814

[8] Yang X Chen W Sun H He C Liu Y Qin M . Baicalin promotes anti-tumor immunity in hepatocellular carcinoma through HIF-1alpha/Lactate/CXCL9 axis. Biochem Pharmacol. (2025) 241:117157. doi: 10.1016/j.bcp.2025.117157. PMID: 40659129

[9] Dong X Liu X Lin D Zhang L Wu Y Chang Y . Baicalin induces cell death of non-small cell lung cancer cells via MCOLN3-mediated lysosomal dysfunction and autophagy blockage. Phytomedicine. (2024) 133:155872. doi: 10.1016/j.phymed.2024.155872. PMID: 39096542

[10] Zhao F Zhao Z Han Y Li S Liu C Jia K . Baicalin suppresses lung cancer growth phenotypes via miR-340-5p/NET1 axis. Bioengineered. (2021) 12:1699–707. doi: 10.1080/21655979.2021.1922052. PMID: 33955315 PMC8806212

[11] Li J Liu H Lin Q Chen H Liu L Liao H . Baicalin suppresses the migration and invasion of breast cancer cells via the TGF-beta/lncRNA-MALAT1/miR-200c signaling pathway. Med (Baltimore). (2022) 101:e29328. doi: 10.1097/MD.0000000000029328. PMID: 36401368 PMC9678613

[12] Wang R Wang C Lu L Yuan F He F . Baicalin and baicalein in modulating tumor microenvironment for cancer treatment: A comprehensive review with future perspectives. Pharmacol Res. (2024) 199:107032. doi: 10.1016/j.phrs.2023.107032. PMID: 38061594

[13] Shao L Zhu L Su R Yang C Gao X Xu Y . Baicalin enhances the chemotherapy sensitivity of oxaliplatin-resistant gastric cancer cells by activating p53-mediated ferroptosis. Sci Rep. (2024) 14:10745. doi: 10.1038/s41598-024-60920-y. PMID: 38730240 PMC11087583

[14] Wei J Liu R Zhang J Liu S Yan D Wen X . Baicalin enhanced oral bioavailability of sorafenib in rats by inducing intestine absorption. Front Pharmacol. (2021) 12:761763. doi: 10.3389/fphar.2021.761763. PMID: 34819863 PMC8606670

[15] Zheng P Xu D Cai Y Zhu L Xiao Q Peng W . A multi-omic analysis reveals that Gamabufotalin exerts anti-hepatocellular carcinoma effects by regulating amino acid metabolism through targeting STAMBPL1. Phytomedicine. (2024) 135:156094. doi: 10.1016/j.phymed.2024.156094. PMID: 39348778

[16] Crowley KE Urben L Hacobian G Geiger KL . Valproic acid for the management of agitation and delirium in the intensive care setting: A retrospective analysis. Clin Ther. (2020) 42:e65–73. doi: 10.1016/j.clinthera.2020.02.007. PMID: 32273047

[17] Vatankhah A Moghaddam SH Afshari S Afshari AR Kesharwani P Sahebkar A . Recent update on anti-tumor mechanisms of valproic acid in glioblastoma multiforme. Pathol Res Pract. (2024) 263:155636. doi: 10.1016/j.prp.2024.155636. PMID: 39395298

[18] Ranco-Juárez EX González-Villasana V Camacho-Moll ME Rendón-Garlant L Ramírez-Flores PN Silva-Ramírez B . Mechanistic insights about sorafenib-, valproic acid- and metformin-induced cell death in hepatocellular carcinoma. Int J Mol Sci. (2024) 25:1760. doi: 10.3390/ijms25031760. PMID: 38339037 PMC10855535

[19] Yang X Liu J Liang Q Sun G . Valproic acid reverses sorafenib resistance through inhibiting activated Notch/Akt signaling pathway in hepatocellular carcinoma. Fundam Clin Pharmacol. (2021) 35:690–9. doi: 10.1111/fcp.12608. PMID: 33015852

[20] Wang X Sun K Dong J Ge Y Liu H Jin X . Carrier-free nanoparticles based on natural products trigger dual "synergy and attenuation" for enhanced phototherapy of liver cancer. Mater Today Bio. (2025) 35:102278. doi: 10.1016/j.mtbio.2025.102278. PMID: 40984881 PMC12450544

[21] Rosini E Pollegioni L . Reactive oxygen species as a double-edged sword: The role of oxidative enzymes in antitumor therapy. Biofactors. (2022) 48:384–99. doi: 10.1002/biof.1789. PMID: 34608689

[22] Li WR Li YK Ren H Guo Z Xiao CL Luo JQ . Lactobacillus reuteri attenuates methotrexate-induced liver injury via modulation of oxidative stress and inflammation through HO-1/GPX4 and NF-κB/NLRP3 pathways. Eur J Pharmacol. (2025) 1006:178185. doi: 10.1016/j.ejphar.2025.178185. PMID: 40992648

[23] Rai V Srivastava A Shekher A Dutta A Gupta SC . Epoxyazadiradione, a neem-derived limonoid exhibits activities against pancreatic cancer through modulation of inflammatory molecules, lncRNAs, ROS, and EMT. Biochim Biophys Acta Gen Subj. (2026) 1870:130938. doi: 10.1016/j.bbagen.2026.130938. PMID: 41881140

[24] Wen RJ Dong X Zhuang HW Pang FX Ding SC Li N . Baicalin induces ferroptosis in osteosarcomas through a novel Nrf2/xCT/GPX4 regulatory axis. Phytomedicine. (2023) 116:154881. doi: 10.1016/j.phymed.2023.154881. PMID: 37209607

[25] Sahasrabudhe SA Terluk MR Kartha RV . N-acetylcysteine pharmacology and applications in rare diseases-repurposing an old antioxidant. Antioxid (Basel). (2023) 12:1316. doi: 10.3390/antiox12071316. PMID: 37507857 PMC10376274

[26] Zhang H Sun K Gao M Xu S . Zinc inhibits lead-induced oxidative stress and apoptosis of ST cells through ROS/PTEN/PI3K/AKT axis. Biol Trace Elem Res. (2024) 202:980–9. doi: 10.1007/s12011-023-03721-0. PMID: 37269454

[27] Donne R Lujambio A . The liver cancer immune microenvironment: Therapeutic implications for hepatocellular carcinoma. Hepatology. (2023) 77:1773–96. doi: 10.1002/hep.32740. PMID: 35989535 PMC9941399

[28] Ma J Wang B Shao H Zhang S Chen X Li F . Hydrogels for localized chemotherapy of liver cancer: a possible strategy for improved and safe liver cancer treatment. Drug Delivery. (2022) 29:1457–76. doi: 10.1080/10717544.2022.2070299. PMID: 35532174 PMC9090357

